# Spatiotemporal dynamics and climate correlates of Parkinson disease mortality among older U.S. adults: national trends and forecasts to 2035

**DOI:** 10.1186/s12883-026-05248-y

**Published:** 2026-08-12

**Authors:** Zhongke Huang, Wenkai Pan, Meng Xia, Gege Zhao, Jian Wang, Hongjie Hu

**Affiliations:** 1 Department of Nuclear Medicine, Sir Run Run Shaw Hospital, Zhejiang University School of Medicine, Hangzhou, 310000 Zhejiang Province China; 2 Department of Radiology, Sir Run Run Shaw Hospital, Zhejiang University School of Medicine, Hangzhou, 310000 Zhejiang Province China

**Keywords:** Parkinson disease, Mortality, Forecasting, Spatial heterogeneity, Climate, Time-series analysis

## Abstract

**Background:**

Parkinson disease (PD) is an increasing cause of mortality among older adults, but national studies have generally examined mortality trends, forecasts, spatial variation, or environmental associations separately. We integrated these components to characterize U.S. PD mortality and its regional climate correlates.

**Methods:**

This ecological time-series study used CDC WONDER mortality data for U.S. adults aged 65 years or older from 1999 to 2024. Joinpoint regression, exponential smoothing state-space forecasting with rolling-origin validation, state-level Moran's I, lagged count models, nonlinear spline analyses, and principal component analysis of regional climate metrics were applied. Nominal and Benjamini-Hochberg-adjusted *P* values were reported.

**Results:**

From 1999 to 2024, PD deaths increased from 14,225 to 39,671, and the age-adjusted mortality rate increased from 41.42 to 71.58 per 100,000 population (average annual percent change, 1.99%; 95% confidence interval [CI], 1.54%-2.45%; *P* < 0.001). The conditional 2035 projection was 91.35 per 100,000 (95% prediction interval, 75.89–110.89). Across 7,502 rolling-origin predictions, mean absolute error was 9.24, root mean squared error was 12.12, mean bias was -6.48, and 95% interval coverage was 51.7%. State-level spatial autocorrelation was not evident in 2024 (Moran's I = 0.022; permutation *P* = 0.326). Primary lag 0–3 temperature estimates did not remain significant after multiplicity correction (adjusted *P* = 0.304), whereas Northeast lag 0–1 mean and apparent temperature estimates remained significant (rate ratios, 0.920 and 0.932 per 10 degrees F; both adjusted *P* = 0.010). No precipitation or sunlight estimate survived correction (minimum adjusted *P* = 0.142 and 0.308, respectively), and 2 of 72 PCA associations remained significant after correction (adjusted *P* = 0.027 and 0.035).

**Conclusions:**

PD mortality increased markedly among older U.S. adults. Forecasts suggested a continued rise but showed limited long-horizon calibration. Climate associations were regional and largely attenuated after multiplicity correction, supporting cautious, hypothesis-generating interpretation.

**Supplementary Information:**

The online version contains supplementary material available at https://doi.org/10.1186/s12883-026-05248-y.

## Introduction

Parkinson disease (PD) is one of the fastest-growing neurological disorders worldwide and is increasingly recognized as a major ageing-related public health challenge. Global burden analyses have shown substantial growth in PD prevalence, disability, and deaths over recent decades, with demographic ageing and population growth expected to further amplify the number of affected individuals in coming decades [1, 2]. Systematic reviews and meta-analyses also indicate that PD prevalence rises steeply with age and varies across populations, underscoring the importance of surveillance systems that can track both temporal change and population heterogeneity [3, 4]. Earlier projections anticipated a marked expansion in the number of people living with PD in populous nations, a pattern now supported by contemporary global burden estimates [5, 6].

Although incidence and prevalence have been widely studied, mortality provides a complementary measure of disease burden because it integrates population ageing, survival, diagnostic practices, comorbidity, and access to care. Mortality trends are unlikely to be spatially uniform: sex, race and ethnicity, age structure, regional context, and state-level differences may all shape the observed burden. For the United States, updated mortality analyses are especially important because recent years include demographic shifts, changes in certification practices, and the disruptive period surrounding the COVID-19 pandemic. A combined framework that quantifies historical changes, identifies subgroup disparities, and projects near-future mortality rates can therefore provide a clearer basis for public health planning than cross-sectional estimates alone.

Environmental context may further modify neurodegenerative disease burden. Large multicountry studies have shown that non-optimal ambient temperature and short-term temperature variability are associated with substantial mortality risks across populations, particularly among older adults and other vulnerable groups [7–9]. For PD specifically, meta-analytic and epidemiological evidence links several environmental exposures with disease risk, including pesticides and solvents, air pollution, and vitamin D or sunlight-related factors [10–14]. These findings suggest that meteorological conditions may be relevant not only as general determinants of mortality but also as contextual exposures that could interact with the vulnerability profile of older populations affected by PD.

PD can contribute to death through progressive neurological disability and systemic complications. Dysphagia and impaired airway protection increase aspiration-pneumonia risk, whereas respiratory dysfunction, immobility, falls, autonomic impairment, frailty, and multimorbidity reduce physiological reserve [31, 32, 34, 35, 39, 40, 49–51]. These pathways may also increase vulnerability to seasonal infection and environmental stress.

However, few studies have integrated long-term PD mortality trends, forward projections, and meteorological correlates within a unified national framework. Existing work on seasonality in PD deaths supports the plausibility of temporal environmental patterning, but the specific roles of temperature, precipitation, sunlight, lag structure, and multivariate climate patterns remain insufficiently characterized [15]. This study analyzed U.S. PD mortality from 1999 to 2024 using Joinpoint regression to quantify temporal changes, exponential smoothing models to forecast mortality rates to 2035, and region-month meteorological models to examine associations between PD monthly crude mortality and temperature, precipitation, and sunlight. Principal component analysis was further applied to summarize correlated climate metrics and evaluate whether integrated climate axes were associated with PD mortality across U.S. Census regions.

Prior U.S. mortality studies have generally not combined updated national surveillance, formal out-of-sample forecast validation, spatial-dependence testing, lag sensitivity, and correlated climate metrics in the same framework [33]. The rationale for the present study was therefore to distinguish robust mortality-surveillance findings from exploratory ecological climate associations.

## Methods

### Study design and data sources

**Fig. 1:**
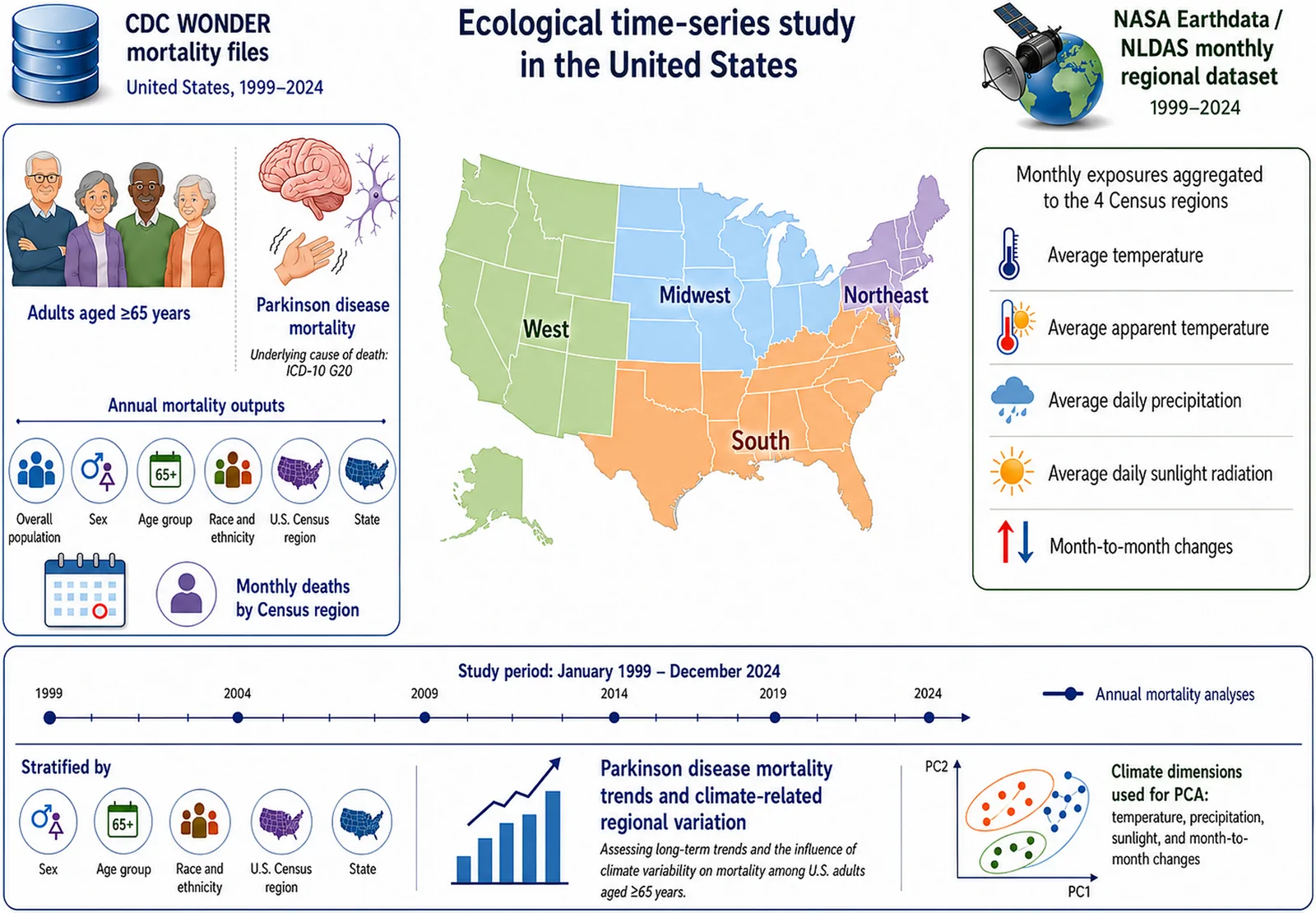
Study design and integrated analytical workflow. CDC WONDER records identified U.S. adults aged 65 years or older with Parkinson disease as the underlying cause of death from 1999 to 2024. Annual data supported trend, subgroup, state, spatial-autocorrelation, and forecast analyses. Monthly Census-region deaths were linked to NASA Earthdata/NLDAS climate metrics for lagged count models, nonlinear analyses, and PCA. ETS, exponential smoothing state space; PCA, principal component analysis

This ecological time-series study evaluated mortality attributed to Parkinson disease (PD) among adults aged 65 years or older in the United States from January 1999 through December 2024. The overall study design and analytic workflow are summarized in Fig. 1. Mortality data were obtained from CDC WONDER, a public online epidemiologic data system that supports access to national vital statistics and related public health datasets [16].

Annual CDC WONDER outputs were extracted for the overall population and by sex, age group, race and ethnicity, U.S. Census region, and state to support long-term trend analyses and mortality forecasting. Monthly PD death counts by Census region were linked to NASA Earthdata/NLDAS meteorological fields for 1999–2024. Spatially contiguous reanalysis meteorological datasets have been used in health analyses to assign population-level ambient exposure metrics across large geographic areas [26]. Age-stratified analyses used groups aged 65–74 years, 75–84 years, and 85 years or older.

NASA Earthdata/NLDAS monthly gridded fields were assigned to U.S. Census regions by using regionmask state polygons and the standard Census region state mapping. Regional exposure values were calculated as area-weighted means using cosine-of-latitude grid weights. Population weighting was not applied. Aggregation used grid cells available within the NLDAS domain.

These area-weighted regional averages represent outdoor ambient conditions rather than individual or population-weighted exposure.

### Mortality outcomes and rate definitions

For long-term trend and forecasting analyses, the primary outcome was the annual age-adjusted mortality rate (AAMR) per 100,000 population. AAMRs and corresponding standard errors and 95% confidence intervals were derived from CDC WONDER outputs and interpreted using established interval-estimation principles for age-adjusted rates [17].

For region-month meteorological analyses, the outcome was the monthly crude PD mortality rate, calculated as monthly PD deaths divided by the corresponding annual Census-region population and multiplied by 100,000. The monthly count models used monthly PD deaths as the dependent variable and included the natural logarithm of annual population as an offset. Seasons were defined as spring (March–May), summer (June–August), autumn (September–November), and winter (December-February). CDC WONDER did not provide monthly age-adjusted mortality rates for the region-month strata used in the meteorological analyses. Monthly crude rates and count models with population offsets were therefore used for these analyses.

### Joinpoint trend analysis

Temporal trends in annual AAMR from 1999 to 2024 were assessed using the Joinpoint Regression Program, Version 5.1.0.0. Trend segments were summarized using annual percent change (APC), and the overall trend during the study period was summarized using average annual percent change (AAPC), consistent with established joinpoint regression and AAPC methods for rate trend analysis [18, 19].

The allowed maximum number of joinpoints was set automatically according to the software-recommended setting for the number of annual observations. Log-linear models were fitted to rates, and the final number and locations of joinpoints were selected using the program-recommended model-selection procedure.

### Forecasting analysis

Mortality rates were projected to 2035 using exponential smoothing state-space models fitted to the natural logarithm of annual AAMR. For each complete time series, three candidate models were considered: a model without trend, a model with an additive trend, and a model with a damped trend. Exponential smoothing and related state-space forecasting approaches have been applied to mortality-rate and epidemiologic time-series forecasting because they accommodate level, trend, and time-series uncertainty in a compact framework [20, 21].

Point forecasts were back-transformed from the log scale to the original AAMR scale. Prediction intervals were estimated using 500 bootstrap simulations from the fitted exponential smoothing model, with the 2.5th and 97.5th percentiles used as the lower and upper bounds of the 95% prediction interval. Forecasts were produced for the overall population, demographic strata, Census regions, race and ethnicity groups, age groups, and states. Candidate ETS specifications were compared using AIC, and the lowest-AIC model was retained for each series. Residual standard deviations on the log scale were summarized as model diagnostics. Bootstrap prediction intervals were generated by simulating 500 future paths from the fitted ETS model.

Forecast validity was additionally assessed using expanding-window rolling-origin evaluation, which prevents future observations from entering model training [52]. Training began with 1999–2008, and subsequent origins were evaluated at horizons up to 11 years. We reported mean absolute error, root mean squared error, mean absolute scaled error, mean bias, observed-predicted correlation, 95% prediction-interval coverage and width, and weighted interval score [53, 54]. The pre-COVID scenario was treated as a sensitivity analysis of training-period dependence rather than a causal estimate of pandemic effects. The validation design and horizon-specific results are summarized in Figure S1.

### Single meteorological exposure analyses

Meteorological analyses were conducted separately for temperature, precipitation, and sunlight radiation. For each exposure block, monthly mortality and meteorological data were merged by Census region, year, and month. Mean temperature and apparent temperature, precipitation, and sunlight radiation metrics were analyzed as monthly climate exposures, following the general time-series framework used in environmental epidemiology [22].

Apparent temperature was calculated as AT = Ta + 0.33 × e—0.70 × ws—4.00, where Ta is air temperature in degrees Celsius, e is vapor pressure in hPa derived from specific humidity and surface pressure, and ws is wind speed in m/s derived from eastward and northward wind components. Apparent temperature values were converted to degrees Fahrenheit for analysis.

Descriptive associations between monthly crude PD mortality rates and meteorological variables were evaluated using Pearson and Spearman correlation coefficients. Scatterplots with seasonal coloring, density overlays, and cubic spline-smoothed curves were used to visualize exposure–response patterns. Seasonal differences in monthly crude mortality rates were assessed by one-way analysis of variance within each Census region.

Region-specific Poisson log-linear models were then fitted to estimate associations between meteorological exposures and monthly PD deaths. Single-lag models included one exposure lag at a time and adjusted for long-term time trend using a natural cubic spline of calendar time and month. Cumulative lag 0–3 models jointly included exposure lags 0 through 3 months to characterize short-term delayed associations, consistent with the distributed-lag modeling framework for environmental time-series data [23, 24].

The 0–3-month window was selected a priori to capture immediate and delayed pathways involving thermal stress, respiratory complications, infection, mobility, and falls while limiting collinearity across 312 monthly observations. Sensitivity analyses compared cumulative lag 0–1, 0–3, and 0–6 windows. Models included calendar-month fixed effects and Newey-West covariance estimates with a 12-month maximum lag [55].

To evaluate potential nonlinearity, cumulative lag 0–3 restricted cubic spline models were fitted for selected mean exposures. In these models, the exposure at each lag from 0 to 3 months was modeled with a natural cubic spline with 3 degrees of freedom, and overall and nonlinear exposure–response tests were used to summarize the cumulative association. Restricted cubic spline functions provide a flexible approach for evaluating dose–response relations without assuming linearity [25].

### Principal component analysis of climate metrics

Principal component analysis (PCA) was used to summarize correlated meteorological variables. Three PCA blocks were constructed: raw monthly climate levels, monthly anomalies, and inter-month changes. Raw monthly levels included average temperature, apparent temperature, precipitation, and sunlight radiation. PCA was used as a dimension-reduction method to represent shared climate variation while reducing collinearity among correlated meteorological inputs [28].

PCA score distributions and loading structures were summarized by Census region. Regional separation in the PC1-PC2 space was evaluated using permutational multivariate analysis of variance with 999 permutations, an approach aligned with non-parametric permutation testing for multivariate association structures [29]. Lagged PC1 variables were created with lags of 0 to 3 months.

Associations between PC1 scores and PD mortality were evaluated with region-specific negative binomial regression models. Models used monthly PD deaths as the dependent variable and the log annual population as an offset. For raw and inter-month change PC1 scores, models adjusted for month and long-term time trend. For monthly anomaly PC1 scores, models adjusted for long-term time trend. Negative binomial models were used to accommodate overdispersion in count and rate data when Poisson variance assumptions may be too restrictive [27].

The NB2 dispersion parameter was estimated from the data. Poisson dispersion and Akaike information criterion values were compared with NB2 results to justify the selected count-data distribution; Newey-West covariance estimates used a 12-month maximum lag [27, 55].

### Spatial analysis, multiplicity, statistical software, and ethics

Annual global Moran's I assessed spatial autocorrelation in state AAMRs using queen-contiguity weights for the 48 contiguous states and 9,999 permutations [56]. Geographic comparisons remained descriptive because this global statistic does not identify local causes. All tests were two-sided. Exact nominal *P* values and Benjamini-Hochberg-adjusted *P* values were reported, with correction families defined by climate domain, across 72 PCA models, and across annual Moran tests [57]. Results with nominal *P* < 0.05 but adjusted *P* > = 0.05 were treated as exploratory. Data processing, forecasting, modeling, and visualization were performed in Python using pandas, NumPy, SciPy, statsmodels, scikit-learn, matplotlib, and seaborn. The analysis used de-identified, publicly available, aggregate mortality data and publicly available meteorological data. Therefore, institutional review board approval and informed consent were not required. The manuscript was prepared in accordance with the Strengthening the Reporting of Observational Studies in Epidemiology (STROBE) reporting guideline for observational studies [30]. Supporting numerical results are organized in Supplementary Tables S1-S11 and Supplementary Data S1-S2.

## Results

**Fig. 2:**
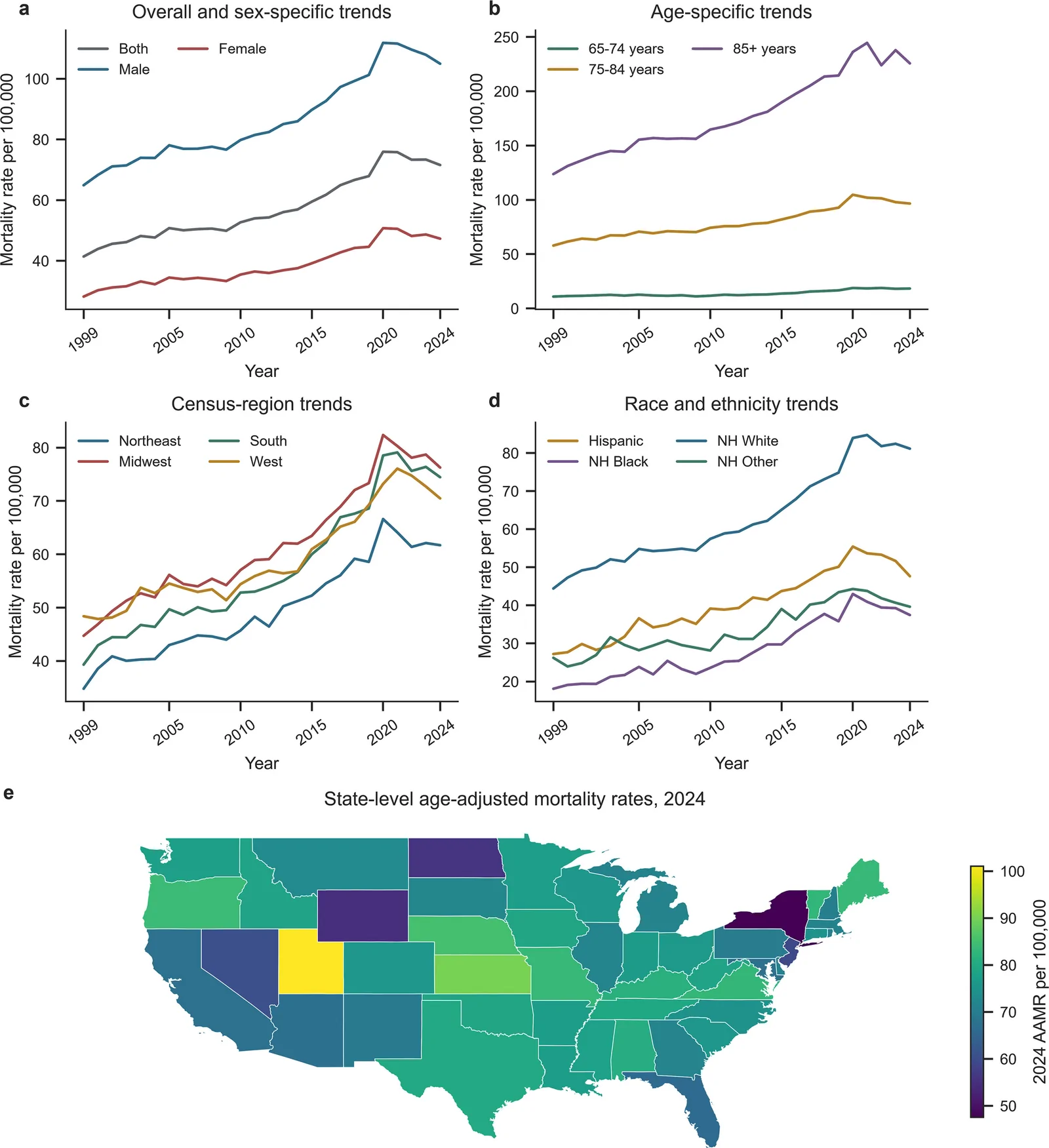
Temporal and geographic patterns of Parkinson disease mortality, 1999–2024. Panels show annual mortality rates overall and by sex (**a**), age group (**b**), Census region (**c**), and race and ethnicity (**d**), followed by 2024 AAMRs for the 48 contiguous states (**e**). Sex, region, race, and state estimates are age adjusted. Age-group estimates are crude rates. Alaska, Hawaii, and the District of Columbia are reported in Table 1 but are not displayed in the contiguous-state map. AAMR, age-adjusted mortality rate; NH, non-Hispanic

From 1999 to 2024, PD mortality increased substantially among U.S. adults aged 65 years or older. The number of deaths increased from 14,225 in 1999 to 39,671 in 2024, corresponding to a 178.88% increase. Over the same period, the AAMR increased from 41.42 (95% CI, 40.74–42.10) to 71.58 (70.88–72.29) per 100,000 population, with an AAPC of 1.99% (95% CI, 1.54–2.45, Table 1 and Fig. 2).

**Table 1:** Parkinson disease mortality trends by demographic and geographic strata, 1999–2024

Measure	Deaths, 1999	Deaths, 2024	Change, %	AAMR, 1999 (95% CI)	AAMR, 2024 (95% CI)	AAPC, % (95% CI)	*P* value
Both	14,225	39,671	178.88	41.42 (40.74 to 42.10)	71.58 (70.88 to 72.29)	1.99 (1.54 to 2.45)	< 0.001
Female	6194	15,145	144.51	28.22 (27.51 to 28.92)	47.31 (46.56 to 48.07)	1.81 (1.26 to 2.36)	< 0.001
Male	8031	24,526	205.39	64.92 (63.47 to 66.36)	104.96 (103.64 to 106.30)	1.75 (1.35 to 2.16)	< 0.001
Midwest	3738	8880	137.56	44.73 (43.30 to 46.17)	76.26 (74.68 to 77.87)	2.10 (1.40 to 2.81)	< 0.001
Northeast	2585	6397	147.47	34.81 (33.47 to 36.15)	61.71 (60.20 to 63.24)	2.01 (1.45 to 2.57)	< 0.001
South	4682	15,623	233.68	39.33 (38.20 to 40.46)	74.45 (73.28 to 75.64)	2.30 (1.77 to 2.84)	< 0.001
West	3220	8771	172.39	48.40 (46.72 to 50.07)	70.48 (69.01 to 71.98)	1.50 (1.05 to 1.96)	< 0.001
Hispanic	377	2397	535.81	27.22 (24.43 to 30.01)	47.65 (45.74 to 49.61)	2.29 (1.69 to 2.89)	< 0.001
NH Black	473	1865	294.29	18.10 (16.46 to 19.74)	37.46 (35.75 to 39.23)	2.81 (2.00 to 3.63)	< 0.001
NH Other	194	1523	685.05	26.22 (22.46 to 29.98)	39.63 (37.66 to 41.68)	1.65 (0.83 to 2.48)	0.000077
NH White	13,154	33,820	157.11	44.41 (43.65 to 45.17)	81.14 (80.27 to 82.01)	2.21 (1.75 to 2.67)	< 0.001
Alabama	198	689	247.98	35.81 (30.82 to 40.81)	80.85 (74.88 to 87.17)	3.46 (3.06 to 3.87)	< 0.001
Alaska	15	40	166.67	NA	48.73 (34.37 to 67.50)	NA	NA
Arizona	352	942	167.61	57.78 (51.71 to 63.85)	67.18 (62.95 to 71.63)	1.09 (0.73 to 1.45)	0.000002
Arkansas	128	398	210.94	34.70 (28.68 to 40.71)	77.16 (69.74 to 85.17)	2.52 (1.27 to 3.80)	0.000076
California	1545	4041	161.55	44.31 (42.10 to 46.52)	67.32 (65.25 to 69.43)	1.95 (1.42 to 2.48)	< 0.001
Colorado	179	631	252.51	45.35 (38.70 to 52.00)	75.93 (70.08 to 82.17)	1.81 (−0.87 to 4.57)	0.187
Connecticut	177	498	181.36	36.06 (30.74 to 41.38)	74.72 (68.29 to 81.60)	2.55 (1.84 to 3.27)	< 0.001
Delaware	37	140	278.38	39.13 (27.55 to 53.94)	71.12 (59.72 to 84.16)	2.40 (1.69 to 3.12)	< 0.001
District of Columbia	20	45	125	28.32 (17.30 to 43.74)	51.51 (37.53 to 69.09)	NA	NA
Florida	1088	3288	202.21	39.47 (37.12 to 41.82)	66.02 (63.78 to 68.32)	1.83 (1.17 to 2.50)	< 0.001
Georgia	259	1093	322.01	35.05 (30.78 to 39.32)	71.63 (67.40 to 76.06)	2.63 (2.03 to 3.24)	< 0.001
Hawaii	71	171	140.85	47.47 (37.07 to 59.88)	54.08 (46.25 to 62.90)	0.30 (−1.94 to 2.60)	0.794
Idaho	66	240	263.64	45.84 (35.45 to 58.32)	78.54 (68.82 to 89.31)	1.78 (1.42 to 2.15)	< 0.001
Illinois	680	1494	119.71	44.90 (41.53 to 48.28)	71.83 (68.22 to 75.58)	1.87 (1.06 to 2.69)	0.000005
Indiana	319	832	160.82	42.90 (38.19 to 47.61)	76.93 (71.76 to 82.37)	1.88 (0.76 to 3.01)	0.000929
Iowa	193	453	134.72	41.11 (35.28 to 46.93)	79.03 (71.89 to 86.71)	2.70 (1.70 to 3.71)	< 0.001
Kansas	166	435	162.05	43.59 (36.93 to 50.24)	90.06 (81.77 to 98.99)	2.93 (1.12 to 4.76)	0.001
Kentucky	177	594	235.59	36.39 (31.03 to 41.76)	81.52 (75.05 to 88.42)	2.49 (1.57 to 3.42)	< 0.001
Louisiana	205	548	167.32	41.87 (36.13 to 47.61)	77.04 (70.68 to 83.83)	2.55 (0.89 to 4.23)	0.002
Maine	84	247	194.05	46.28 (36.91 to 57.30)	83.62 (73.46 to 94.84)	2.25 (1.78 to 2.71)	< 0.001
Maryland	257	675	162.65	45.15 (39.63 to 50.68)	67.25 (62.25 to 72.54)	1.67 (1.35 to 1.99)	< 0.001
Massachusetts	420	872	107.62	46.89 (42.40 to 51.38)	71.39 (66.71 to 76.30)	1.75 (1.09 to 2.41)	< 0.001
Michigan	505	1253	148.12	42.55 (38.83 to 46.26)	71.40 (67.47 to 75.50)	2.08 (1.14 to 3.04)	0.000014
Minnesota	402	746	85.57	64.09 (57.80 to 70.37)	77.30 (71.84 to 83.09)	0.67 (−0.06 to 1.41)	0.072
Mississippi	115	361	213.91	34.21 (27.95 to 40.47)	77.84 (69.96 to 86.38)	3.57 (2.77 to 4.38)	< 0.001
Missouri	309	886	186.73	40.63 (36.10 to 45.16)	83.36 (77.94 to 89.06)	2.79 (2.48 to 3.11)	< 0.001
Montana	55	150	172.73	45.42 (34.22 to 59.12)	72.86 (61.60 to 85.64)	1.76 (1.16 to 2.37)	0.000003
Nebraska	118	270	128.81	47.61 (38.98 to 56.23)	85.09 (75.20 to 95.93)	2.67 (2.26 to 3.08)	< 0.001
Nevada	69	303	339.13	39.52 (30.57 to 50.28)	60.56 (53.83 to 67.93)	2.19 (1.64 to 2.73)	< 0.001
New Hampshire	64	188	193.75	44.27 (34.09 to 56.53)	70.46 (60.70 to 81.39)	2.57 (1.96 to 3.18)	< 0.001
New Jersey	428	938	119.16	38.72 (35.05 to 42.39)	59.33 (55.59 to 63.27)	1.71 (1.43 to 1.99)	< 0.001
New Mexico	83	266	220.48	42.71 (34.02 to 52.95)	69.33 (61.20 to 78.26)	0.69 (0.20 to 1.18)	0.008
New York	575	1694	194.61	23.64 (21.71 to 25.58)	47.54 (45.30 to 49.87)	2.85 (2.51 to 3.19)	< 0.001
North Carolina	347	1282	269.45	38.37 (34.33 to 42.42)	74.48 (70.43 to 78.71)	2.81 (2.53 to 3.10)	< 0.001
North Dakota	49	74	51.02	47.93 (35.34 to 63.55)	55.87 (43.78 to 70.41)	0.59 (−1.96 to 3.20)	0.655
Ohio	630	1546	145.4	42.60 (39.28 to 45.93)	76.44 (72.66 to 80.37)	2.16 (1.40 to 2.94)	< 0.001
Oklahoma	177	489	176.27	39.38 (33.58 to 45.19)	79.04 (72.16 to 86.42)	3.29 (0.53 to 6.13)	0.019
Oregon	249	632	153.82	55.41 (48.53 to 62.30)	84.06 (77.59 to 90.95)	1.57 (1.00 to 2.15)	< 0.001
Pennsylvania	734	1712	133.24	37.85 (35.11 to 40.58)	69.71 (66.44 to 73.10)	2.38 (1.26 to 3.51)	0.000027
Rhode Island	54	140	159.26	33.30 (25.02 to 43.45)	70.40 (59.20 to 83.13)	2.96 (2.12 to 3.81)	< 0.001
South Carolina	168	704	319.05	37.56 (31.86 to 43.25)	75.89 (70.31 to 81.81)	2.68 (2.30 to 3.07)	< 0.001
South Dakota	39	112	187.18	33.95 (24.14 to 46.41)	72.33 (59.46 to 87.24)	2.08 (1.43 to 2.74)	0.000001
Tennessee	212	885	317.45	31.47 (27.23 to 35.71)	79.39 (74.20 to 84.87)	3.12 (2.05 to 4.21)	< 0.001
Texas	859	3002	249.48	44.04 (41.09 to 46.99)	79.92 (77.06 to 82.85)	2.17 (1.56 to 2.79)	< 0.001
Utah	106	365	244.34	58.73 (47.54 to 69.92)	101.07 (90.87 to 112.15)	2.19 (0.37 to 4.03)	0.018
Vermont	49	108	120.41	63.65 (47.09 to 84.14)	82.72 (67.78 to 100.08)	1.58 (0.80 to 2.37)	0.000326
Virginia	339	1155	240.71	45.70 (40.83 to 50.58)	82.98 (78.25 to 87.94)	2.23 (1.49 to 2.98)	< 0.001
Washington	407	937	130.22	60.95 (55.03 to 66.88)	77.55 (72.63 to 82.72)	1.04 (0.51 to 1.57)	0.000107
West Virginia	96	275	186.46	35.90 (29.08 to 43.84)	78.69 (69.62 to 88.65)	3.35 (2.82 to 3.89)	< 0.001
Wisconsin	328	779	137.5	45.27 (40.37 to 50.17)	76.14 (70.86 to 81.72)	2.24 (1.16 to 3.33)	0.000044
Wyoming	23	53	130.43	42.59 (27.00 to 63.90)	54.76 (40.95 to 71.85)	NA	NA
65–74 years	2009	6505	223.79	10.91 (10.91 to 10.91)	18.35 (18.35 to 18.35)	1.90 (1.28 to 2.52)	< 0.001
75–84 years	7077	18,643	163.43	57.89 (57.89 to 57.89)	96.60 (96.60 to 96.60)	1.89 (1.44 to 2.35)	< 0.001
85 + years	5139	14,523	182.6	123.71 (123.71 to 123.71)	225.68 (225.68 to 225.68)	2.22 (1.73 to 2.72)	< 0.001

Abbreviations: AAMR, age-adjusted mortality rate; AAPC, average annual percent change; CI, confidence interval; NA, not available. NA indicates that the estimate was not reported because the corresponding rate or Joinpoint trend estimate was unreliable or not estimable from the available death counts. An asterisk indicates statistical significance in the original Joinpoint output

### Long-term mortality trends and subgroup patterns

Mortality was higher in men than in women throughout the study period. In 2024, the AAMR was 104.96 (95% CI, 103.64–106.30) per 100,000 in men and 47.31 (46.56–48.07) per 100,000 in women. Deaths increased by 205.39% in men and 144.51% in women, whereas both groups showed significant upward AAPC estimates (men, 1.75%, women, 1.81%, Table 1).

All four U.S. Census regions showed significant increases in PD mortality. The South had the fastest increase, with an AAPC of 2.30% (95% CI, 1.77–2.84), followed by the Midwest (2.10%), Northeast (2.01%), and West (1.50%). In 2024, the Midwest had the highest regional AAMR, 76.26 (95% CI, 74.68–77.87) per 100,000, followed by the South, West, and Northeast (Table 1 and Fig. 2).

Race and ethnicity patterns also differed. Non-Hispanic White adults had the highest 2024 AAMR, 81.14 (95% CI, 80.27–82.01) per 100,000, whereas non-Hispanic Black adults had the highest AAPC, 2.81% (95% CI, 2.00–3.63). Mortality increased steeply with age: in 2024, the crude death rate was 18.35 per 100,000 among adults aged 65–74 years, 96.60 per 100,000 among those aged 75–84 years, and 225.68 per 100,000 among those aged 85 years or older (Table 1 and Fig. 2F). State-level patterns were heterogeneous, with the highest 2024 AAMRs observed in Utah, Kansas, Nebraska, Oregon, Maine, Missouri, Virginia, and Vermont (Table 1 and Fig. 2A and C).

Annual Moran's I was intermittently positive earlier in the study period but decreased to 0.022 in 2024 (permutation *P* = 0.326; adjusted *P* = 0.326; Figure S2). The latest state map was therefore interpreted descriptively rather than as evidence of current global clustering (Figure S3). Annual estimates are provided in Supplementary Table S8.

### Forecasted mortality rates through 2035

**Fig. 3:**
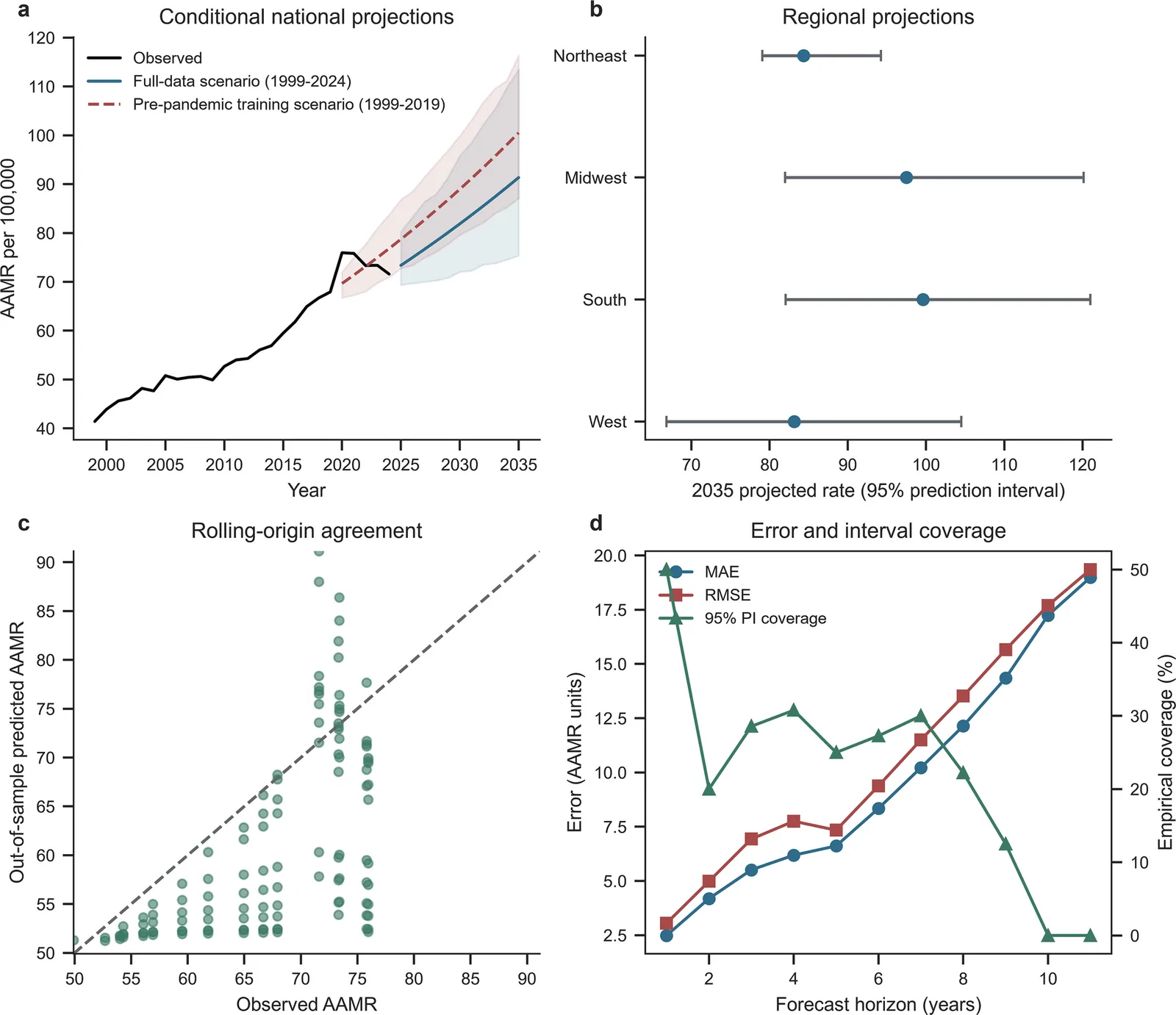
Conditional forecasts to 2035 and rolling-origin validation. Observed national AAMR and two ETS projections are shown with 95% prediction intervals (**a**): the primary full-data scenario used 1999–2024 observations, including the COVID-19 period, whereas the pre-pandemic training sensitivity scenario used 1999–2019 observations. Full-data 2035 projections are shown by Census region (**b**). Rolling-origin predictions are compared with observed AAMRs (**c**), and MAE, RMSE, and empirical 95% prediction-interval coverage are shown by horizon (**d**). The scenario contrast measures training-period sensitivity and is not a causal estimate of pandemic effects. AAMR, age-adjusted mortality rate; ETS, exponential smoothing state space; MAE, mean absolute error; RMSE, root mean squared error

ETS forecasting suggested that PD mortality rates will continue to rise through 2035 (Fig. 3). Under the full-data scenario, the overall AAMR was projected to reach 91.35 (95% prediction interval, 75.89–110.89) per 100,000 by 2035. Projected rates remained higher in men than in women, with 2035 estimates of 129.67 (108.30–157.16) per 100,000 in men and 59.91 (49.47–73.77) per 100,000 in women. (Supplementary Tables S1 and S2).

The age gradient persisted in forecasts. Under the full-data scenario, the 2035 AAMR was projected to be 302.39 (95% prediction interval, 256.26–353.55) per 100,000 among adults aged 85 years or older, compared with 121.78 (102.78–146.52) among those aged 75–84 years and 23.13 (17.57–30.14) among those aged 65–74 years. Regional projections were highest in the South and Midwest. State-level forecasts identified Tennessee, Nebraska, Mississippi, West Virginia, Alabama, Rhode Island, Kentucky, and Iowa among the highest projected states in the full-data scenario.

The pre-COVID scenario yielded a higher projected overall AAMR of 100.52 (95% prediction interval, 87.51–115.23) per 100,000 by 2035, suggesting that forecasts were sensitive to whether post-2019 years were included. In this scenario, projected rates were also higher for men, adults aged 85 years or older, non-Hispanic White adults, and several Midwest and South states (Fig. 3).

Across 62 complete series and 7,502 rolling-origin predictions, pooled mean absolute error was 9.24, root mean squared error was 12.12, mean absolute scaled error was 3.13, and mean bias was −6.48 AAMR units. Empirical 95% interval coverage was 51.7%, mean interval width was 17.30, and mean weighted interval score was 5.85. At the one-year horizon, mean absolute error was 4.40, root mean squared error was 5.94, bias was −1.24, and coverage was 75.6% (Fig. 3 and Figure S1). Errors and negative bias increased with horizon, supporting conditional rather than deterministic interpretation of the 2035 projections. Complete validation summaries are provided in Supplementary Table S7, with prediction-level output in Supplementary Data S1.

### Temperature and apparent temperature associations

**Fig. 4:**
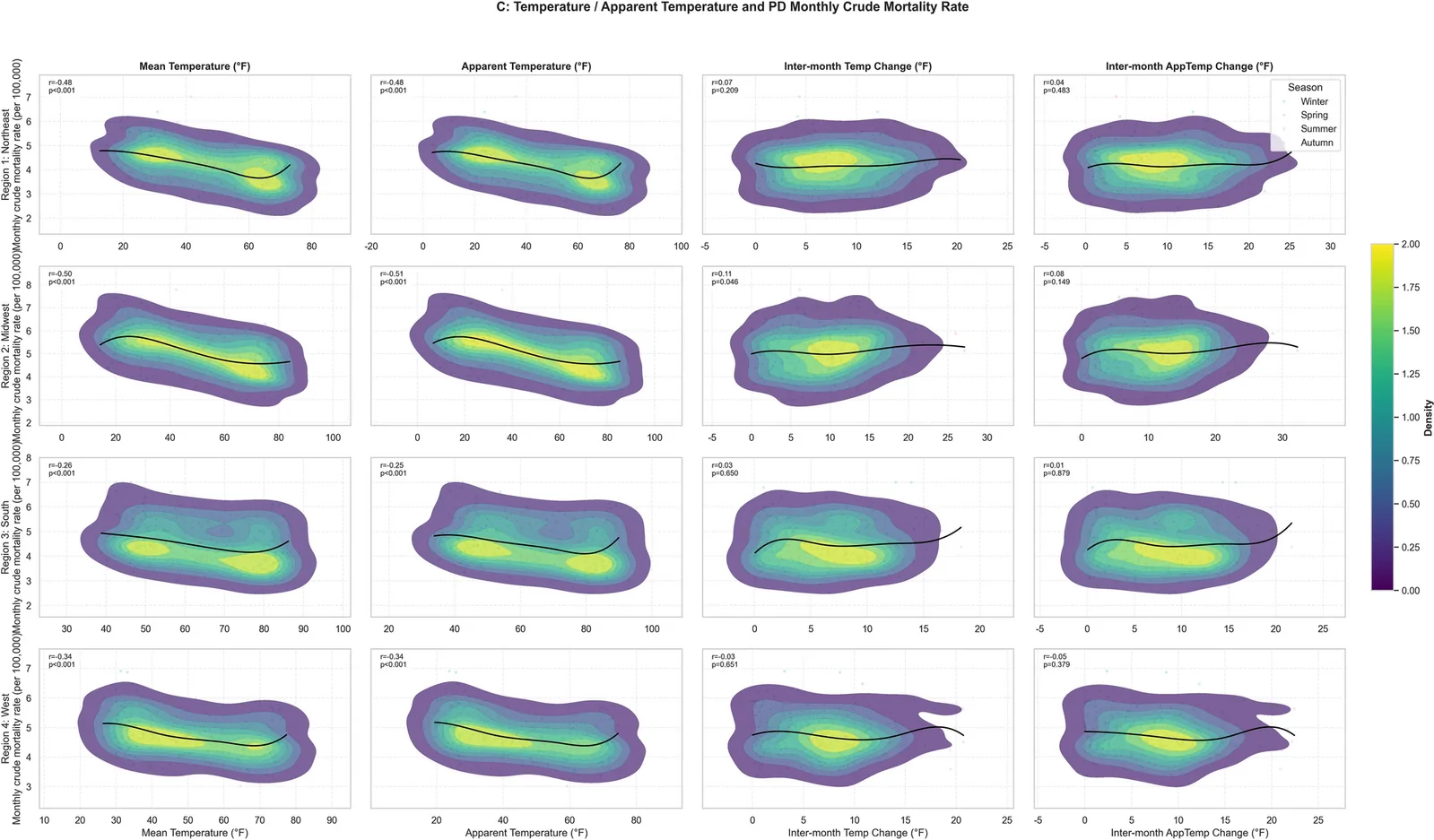
Descriptive temperature and apparent-temperature associations. Rows represent Census regions and columns represent mean temperature, apparent temperature, inter-month temperature change, and inter-month apparent-temperature change. Points are region-month observations colored by season, filled contours show joint observation density, and black curves are descriptive spline smooths. Annotations report Spearman correlations and nominal *P* values. These unadjusted plots describe seasonal patterning and are not interpreted as causal effects

Temperature metrics showed strong descriptive inverse correlations with monthly crude PD mortality, especially in the Midwest and Northeast (Fig. 4). The strongest Pearson correlations were observed for Midwest apparent temperature (r = −0.518), Midwest mean temperature (r = −0.515), Northeast mean temperature (r = −0.493), and Northeast apparent temperature (r = −0.491), all with very small *P* values. (Supplementary Table S3).

**Fig. 5:**
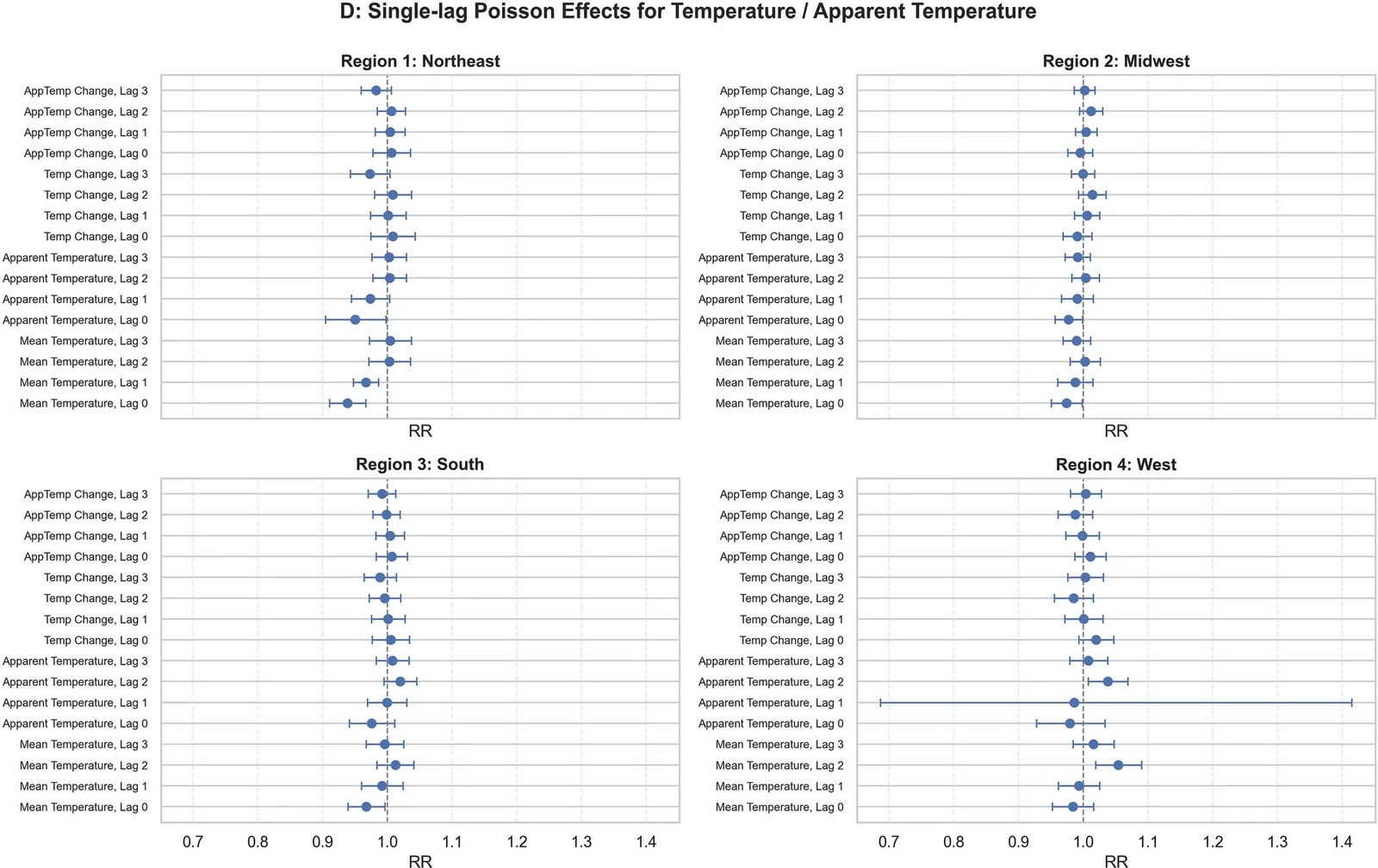
Single-lag temperature and apparent-temperature estimates. Regional forest plots show RRs and 95% CIs at lags 0, 1, 2, and 3 for mean temperature, apparent temperature, and their inter-month changes. Models use monthly deaths with a population offset, calendar-month fixed effects, a centered natural cubic spline of time, and Newey-West covariance estimates. Temperature effects are scaled per 10 degrees F. CI, confidence interval; RR, rate ratio

These descriptive patterns largely reflected seasonality and were not interpreted as adjusted effects. The seasonal mortality distribution is shown in Figure S4. The original regional time series and monthly temperature profiles are retained together in Figure S5. Density and smoothed descriptive temperature panels are shown in Fig. 4, and the single-lag temperature forest plots are shown in Fig. 5.

Adjusted lagged Poisson models showed region- and lag-specific associations. In the Northeast, mean temperature was associated with lower PD mortality at lag 0 (RR per + 10 degrees Fahrenheit, 0.938, 95% CI, 0.911–0.967) and lag 1 (RR, 0.967, 95% CI, 0.948–0.987). In contrast, the West showed positive delayed associations at lag 2 for mean temperature (RR, 1.054, 95% CI, 1.019–1.090) and apparent temperature (RR, 1.038, 95% CI, 1.008–1.069). Cumulative lag 0–3 effects were significant only in the Northeast for mean temperature (RR, 0.938, 95% CI, 0.883–0.996) and apparent temperature (RR, 0.945, 95% CI, 0.897–0.997). The complete set of single-lag estimates is shown in Fig. 5.

**Fig. 6:**
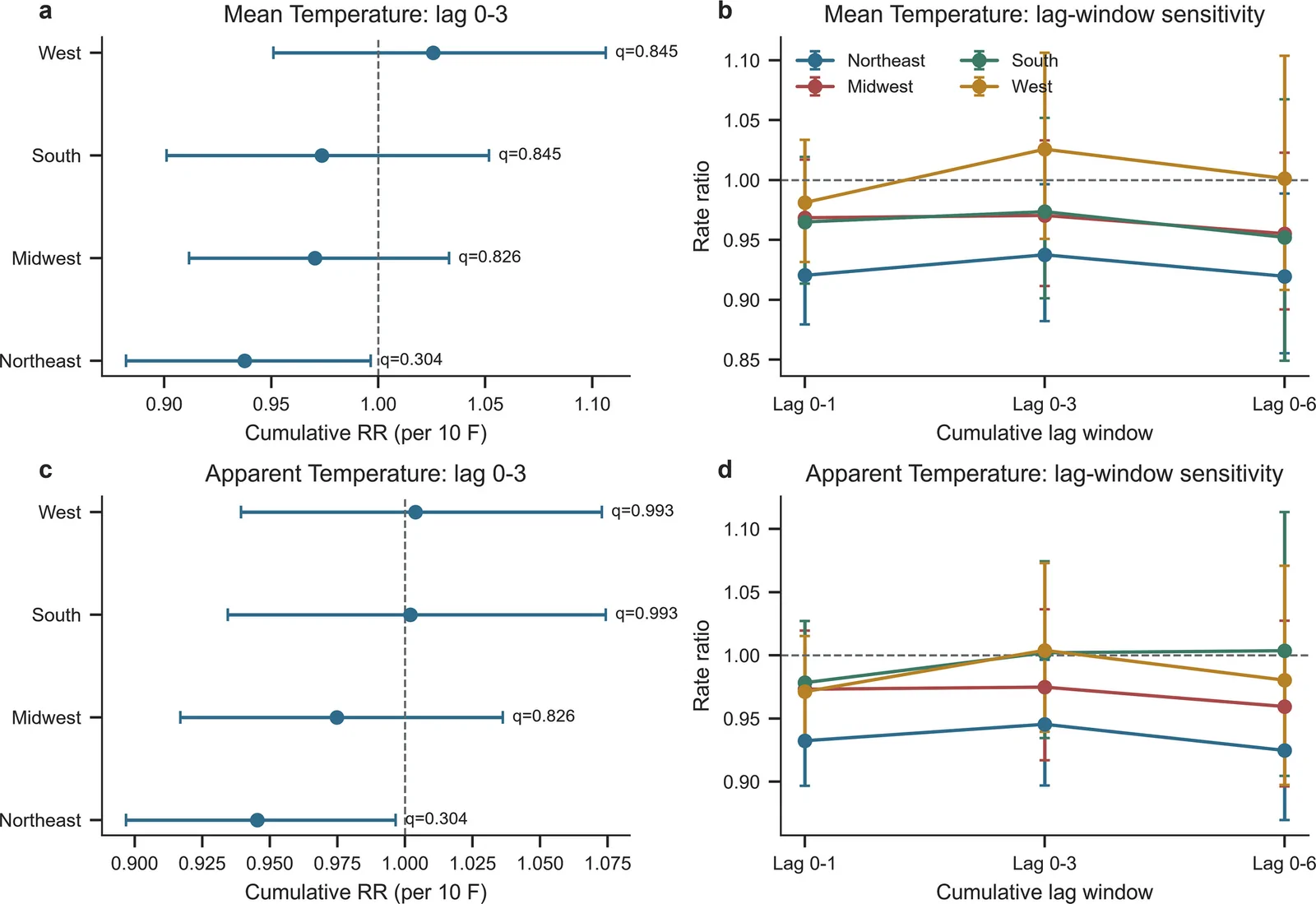
Temperature associations and lag-window sensitivity. Region-specific Poisson models estimated cumulative RRs for mean temperature (**a**) and apparent temperature (**c**) at lag 0–3. Corresponding lag 0–1, 0–3, and 0–6 sensitivity estimates are shown in b and d. Models included calendar-month fixed effects, a centered natural cubic spline of time, a log-population offset, and Newey-West covariance estimates. Points and bars are RRs and 95% CIs per 10 degrees F. Adjusted *P* values used BH correction across the temperature-domain lag-window family. BH, Benjamini-Hochberg; CI, confidence interval; RR, rate ratio

These single-lag findings were nominal. In the prespecified cumulative lag 0–3 models, Northeast mean and apparent temperature estimates had adjusted *P* = 0.304 and did not remain significant. In sensitivity analysis, the Northeast lag 0–1 estimates remained significant after correction (mean temperature RR, 0.920; apparent temperature RR, 0.932; both adjusted *P* = 0.010; Fig. 6). Correction-family summaries and test-level adjusted results are provided in Supplementary Table S9 and Supplementary Data S2; alternative lag-window estimates are provided in Supplementary Table S10.

**Fig. 7:**
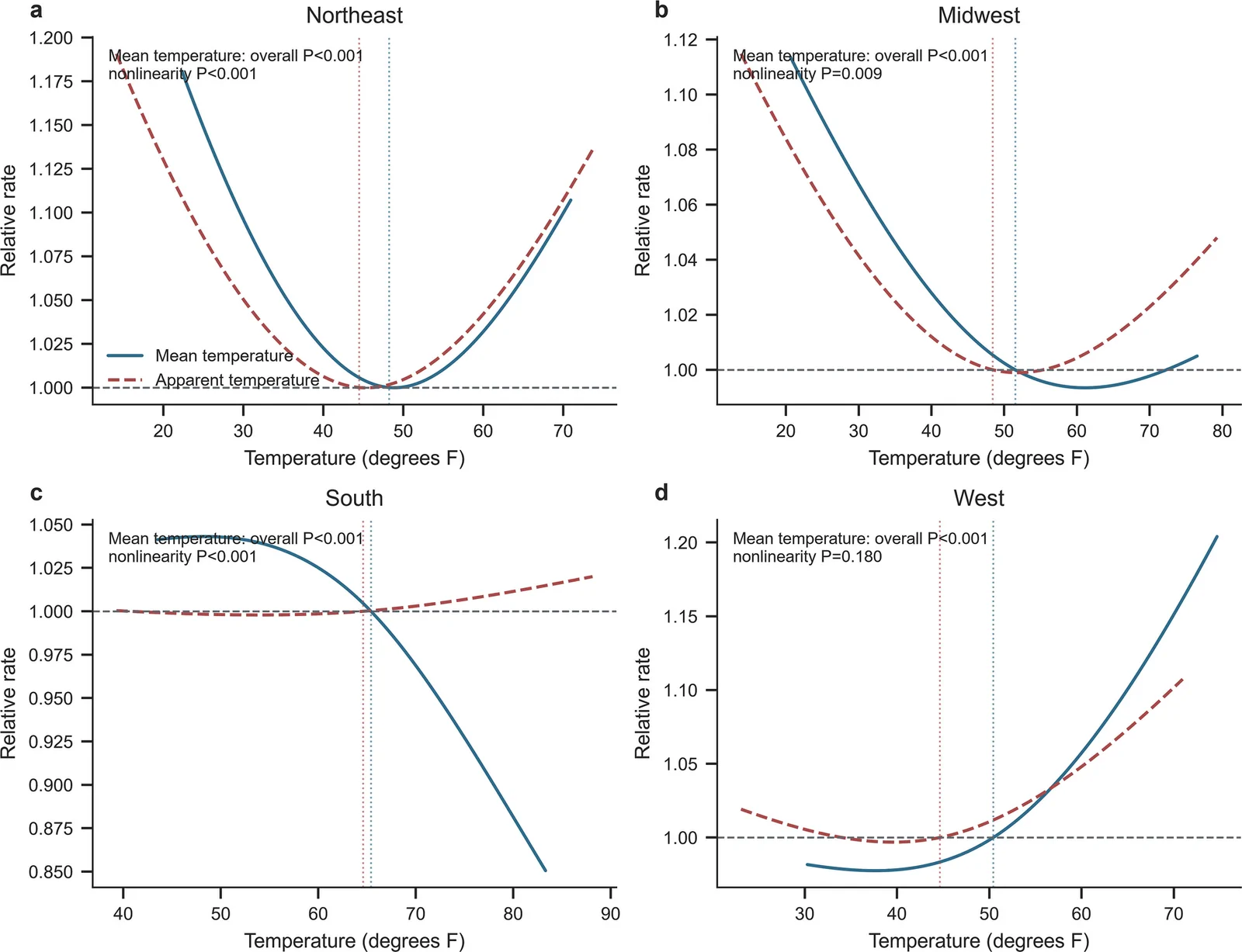
Nonlinear temperature and apparent-temperature associations. Region-specific cumulative lag 0–3 natural cubic-spline curves are overlaid for mean temperature and apparent temperature in the Northeast (**a**), Midwest (**b**), South (**c**), and West (**d**). Relative rates are referenced to the region-specific median exposure; the horizontal dashed line denotes a relative rate of 1. Curves are exploratory estimates adjusted for calendar month, long-term trend, and population offset and should not be interpreted as confirmatory causal effects

Cumulative lag 0–3 restricted cubic spline models supported nonlinear exposure–response patterns for temperature and apparent temperature. Overall spline tests were significant in all eight region-exposure curves, and nonlinearity was detected in six of eight curves, mainly in the Northeast, Midwest, and South (Fig. 7).

These nonlinear results were exploratory and were not treated as confirmatory causal effects after accounting for the broader multiplicity of climate analyses (Fig. 7).

### Precipitation associations

**Fig. 8:**
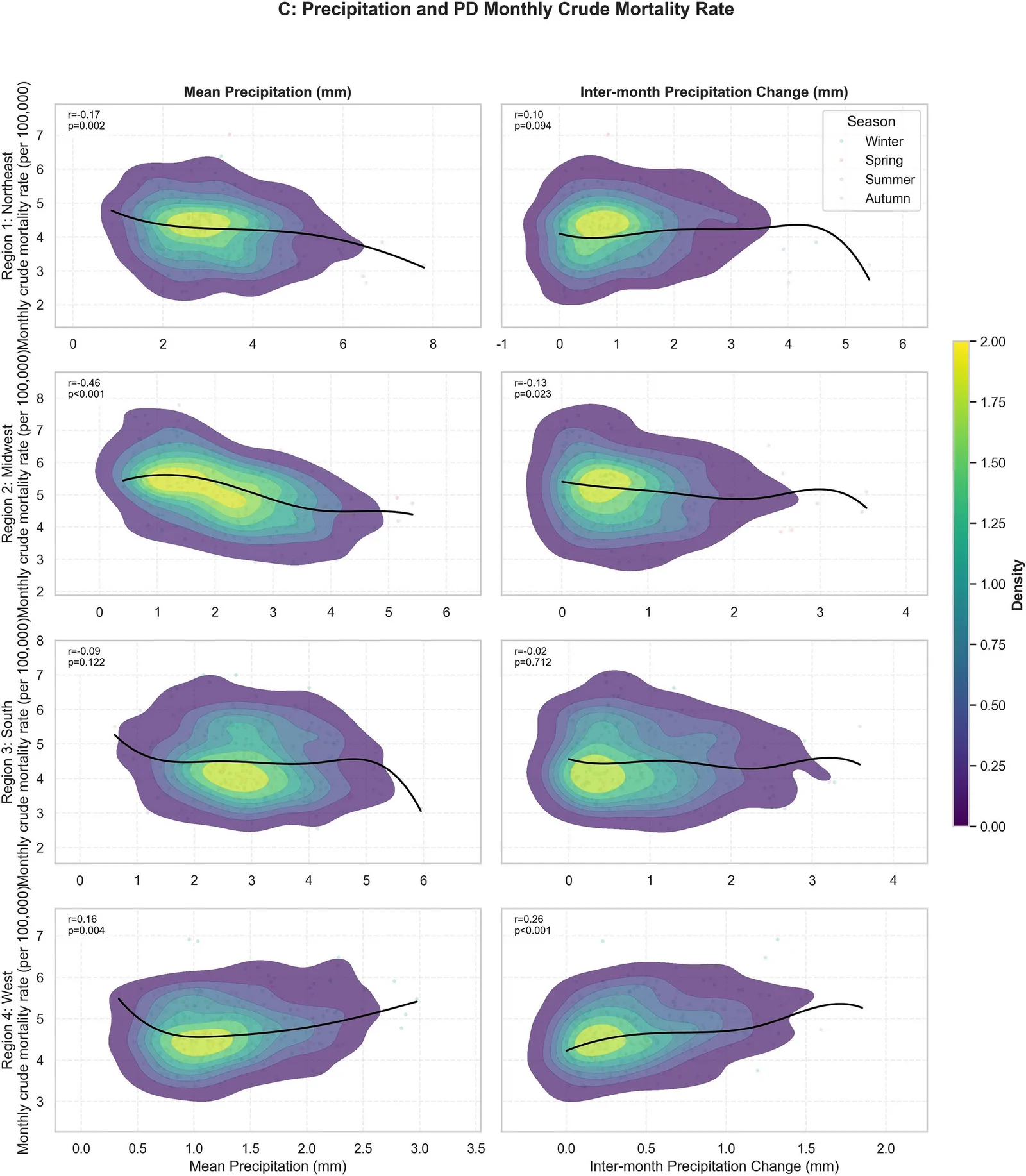
Descriptive precipitation-mortality associations. Rows represent Census regions and columns represent mean precipitation and inter-month precipitation change. Points are region-month observations colored by season, filled contours show joint observation density, and black curves are descriptive spline smooths. Annotations report Spearman correlations and nominal *P* values. These unadjusted plots are descriptive

Precipitation showed weaker and more heterogeneous associations with PD mortality than temperature (Fig. 8). Descriptive correlations were strongest for Midwest mean precipitation (Pearson r = −0.457), West inter-month precipitation change (r = 0.274), West mean precipitation (r = 0.200), and Northeast mean precipitation (r = −0.190). (Supplementary Table S4) The original precipitation time-series and monthly-profile panels are retained together in Figure S6.

**Fig. 9:**
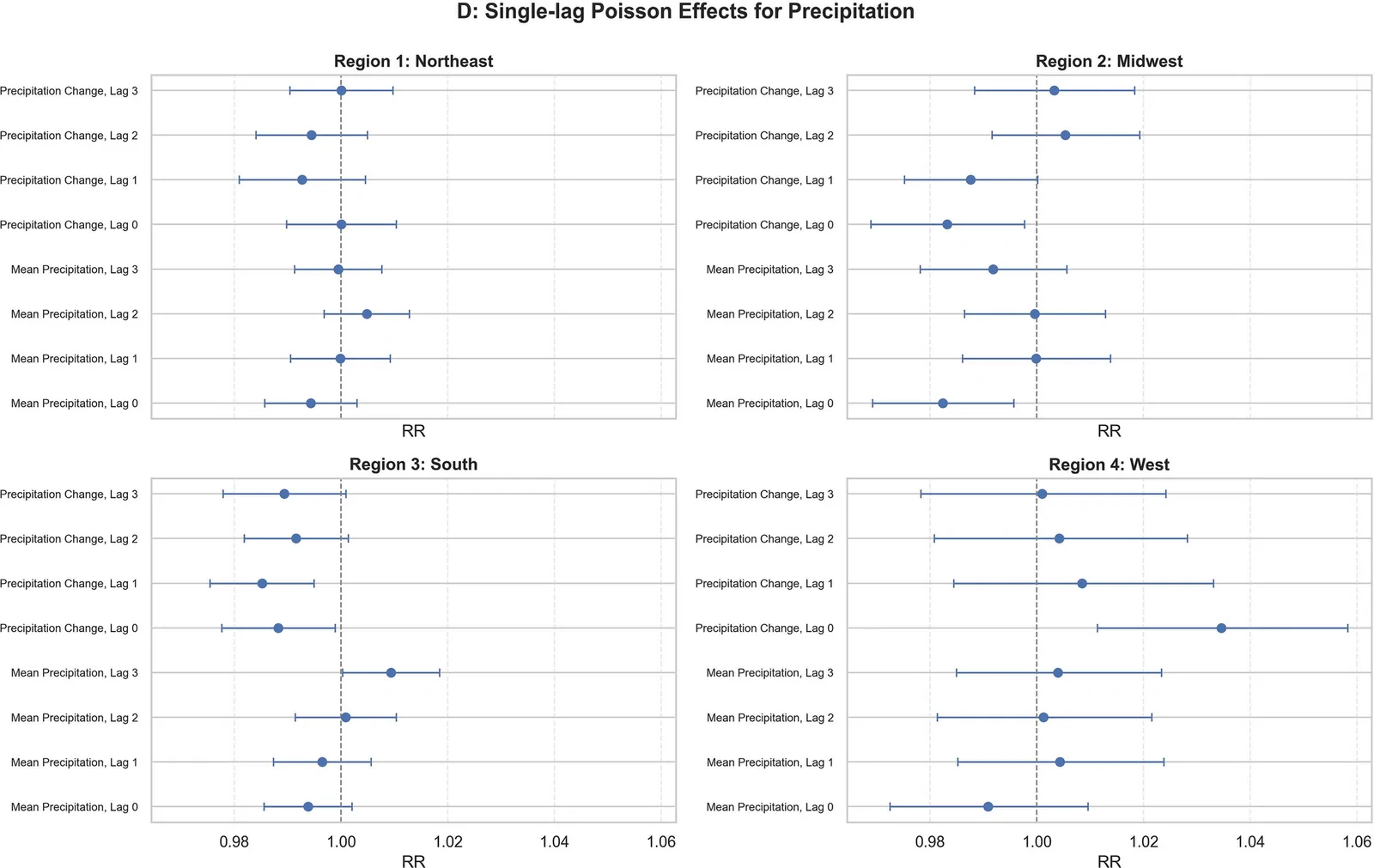
Single-lag precipitation estimates. Regional forest plots show RRs and 95% CIs at lags 0, 1, 2, and 3 for mean precipitation and inter-month precipitation change. Models use monthly deaths with a population offset, calendar-month fixed effects, a centered natural cubic spline of time, and Newey-West covariance estimates. Effects are scaled per 1 mm. CI, confidence interval; RR, rate ratio

In adjusted Poisson models, significant single-lag associations were observed for South precipitation change at lag 1 (RR per + 1 mm, 0.985, 95% CI, 0.975–0.995), West precipitation change at lag 0 (RR, 1.035, 95% CI, 1.011–1.058), Midwest mean precipitation at lag 0 (RR, 0.982, 95% CI, 0.969–0.996), and South mean precipitation at lag 3 (RR, 1.009, 95% CI, 1.000–1.018). Cumulative lag 0–3 models showed inverse associations for South precipitation change (RR, 0.970, 95% CI, 0.953–0.987) and Midwest mean precipitation (RR, 0.975, 95% CI, 0.960–0.990). The complete set of single-lag estimates is shown in Fig. 9, and lag-window sensitivity is shown in Figure S7.

Restricted cubic spline analyses suggested nonlinear precipitation-mortality relationships in selected regions. Overall tests were significant in three of four regional mean-precipitation curves, and nonlinearity was detected in the Northeast and Midwest (Figure S8).

No precipitation lag-window association remained significant after BH correction; the minimum adjusted *P* value was 0.142. Detailed descriptive and single-lag results are shown in Figs. 8 and 9, respectively. Nonlinear precipitation curves are included in Figure S8 and are interpreted as exploratory.

### Sunlight radiation associations

**Fig. 10:**
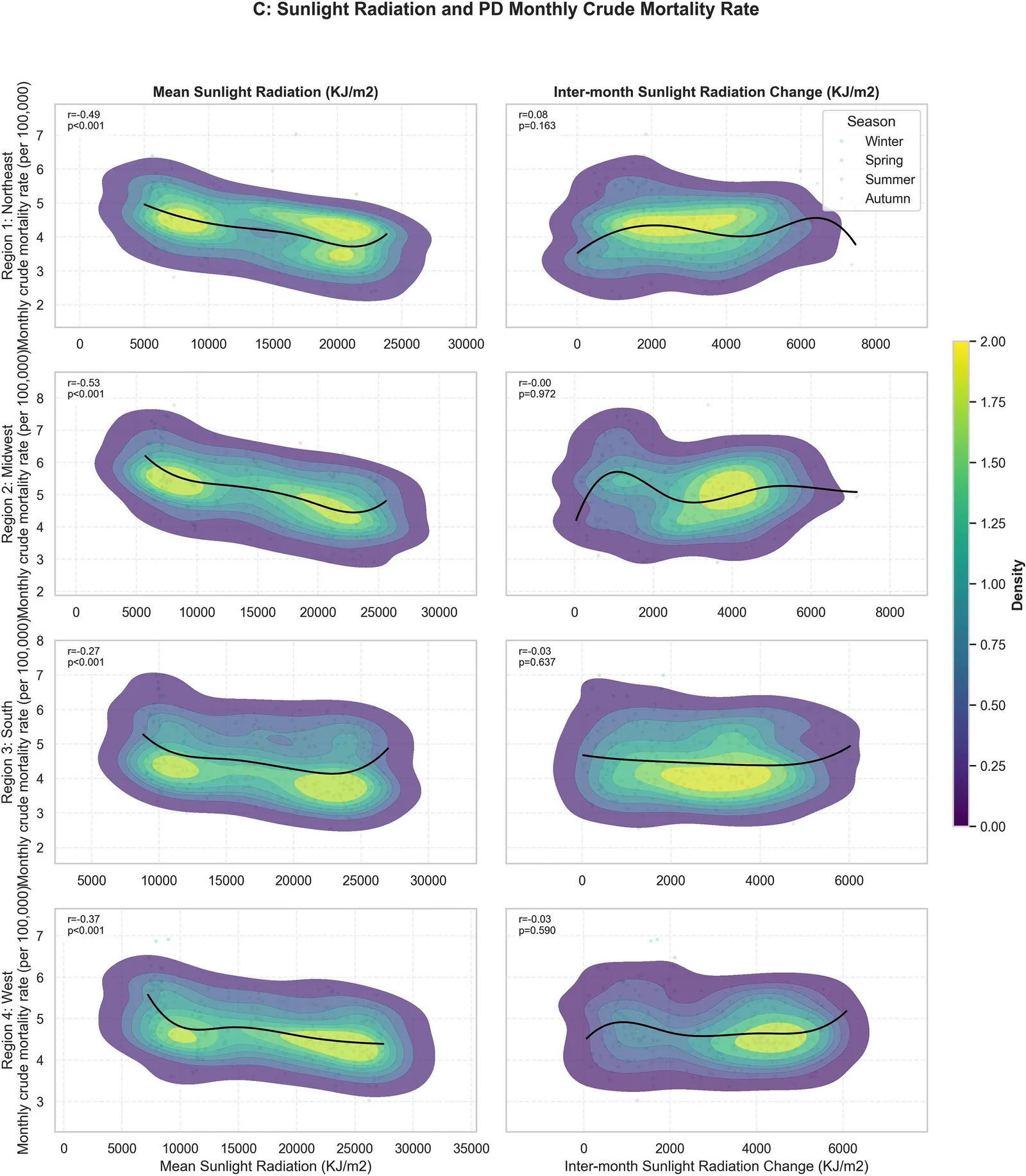
Descriptive sunlight-radiation and mortality associations. Rows represent Census regions and columns represent mean sunlight radiation and inter-month sunlight-radiation change. Points are region-month observations colored by season, filled contours show joint observation density, and black curves are descriptive spline smooths. Annotations report Spearman correlations and nominal *P* values. These unadjusted plots are descriptive

**Fig. 11:**
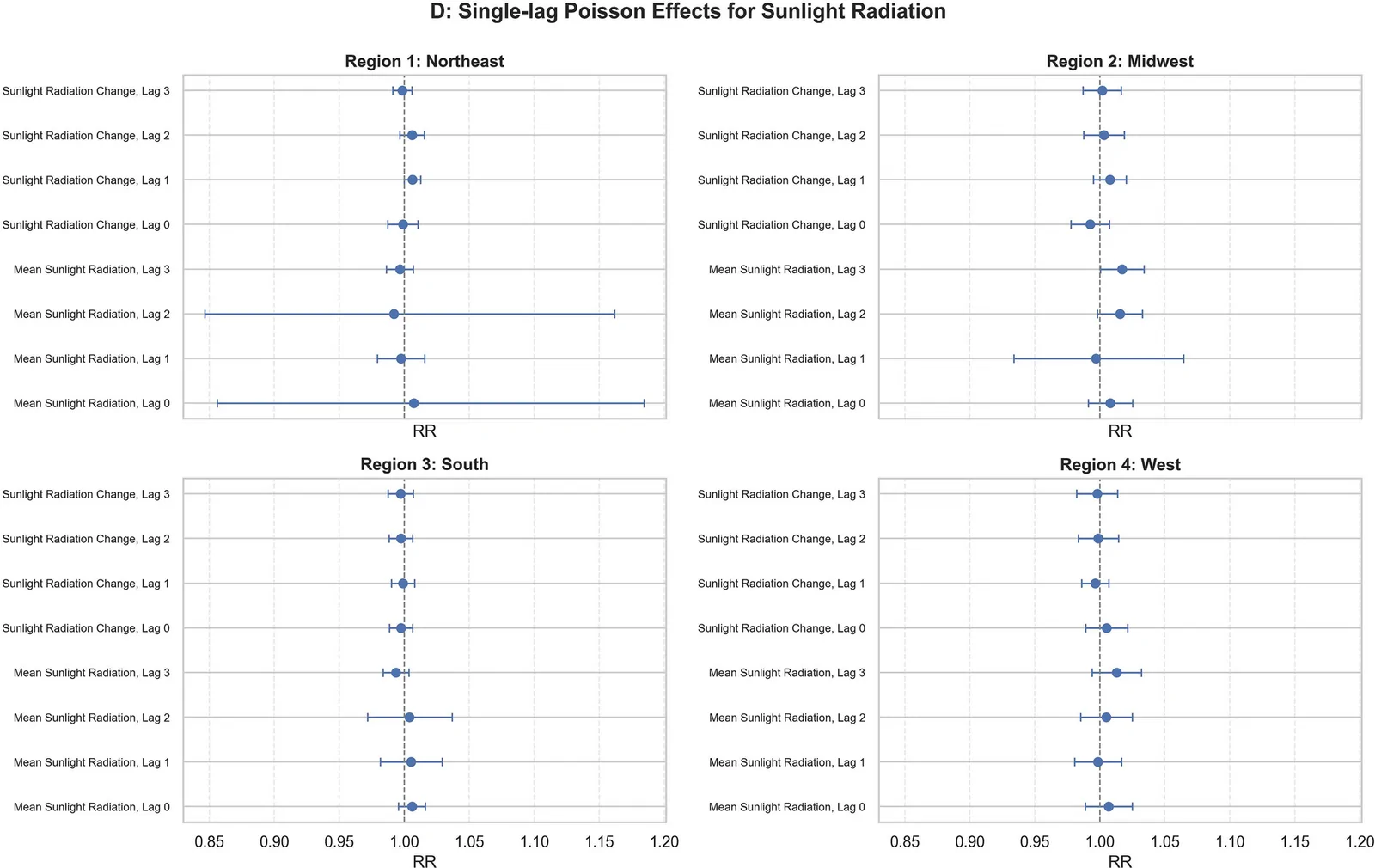
Single-lag sunlight-radiation estimates. Regional forest plots show RRs and 95% CIs at lags 0, 1, 2, and 3 for mean sunlight radiation and inter-month sunlight-radiation change. Models use monthly deaths with a population offset, calendar-month fixed effects, a centered natural cubic spline of time, and Newey-West covariance estimates. Effects are scaled per 1,000 kJ/m2. CI, confidence interval; RR, rate ratio

Mean daily sunlight radiation was inversely correlated with monthly crude PD mortality in all four Census regions, with the strongest correlations in the Midwest (Pearson r = −0.535) and Northeast (r = −0.485, Fig. 10). After adjustment for time trend, calendar month, and population offset, significant single-lag findings were limited: Midwest mean sunlight radiation at lag 3 was associated with higher mortality (RR per + 1,000 kJ/m2, 1.017, 95% CI, 1.000–1.034), and Northeast sunlight radiation change at lag 1 had an RR of 1.006 (95% CI, 1.000–1.013). (Supplementary Table S5) The complete set of single-lag sunlight estimates is shown in Fig. 11.

**Fig. 12:**
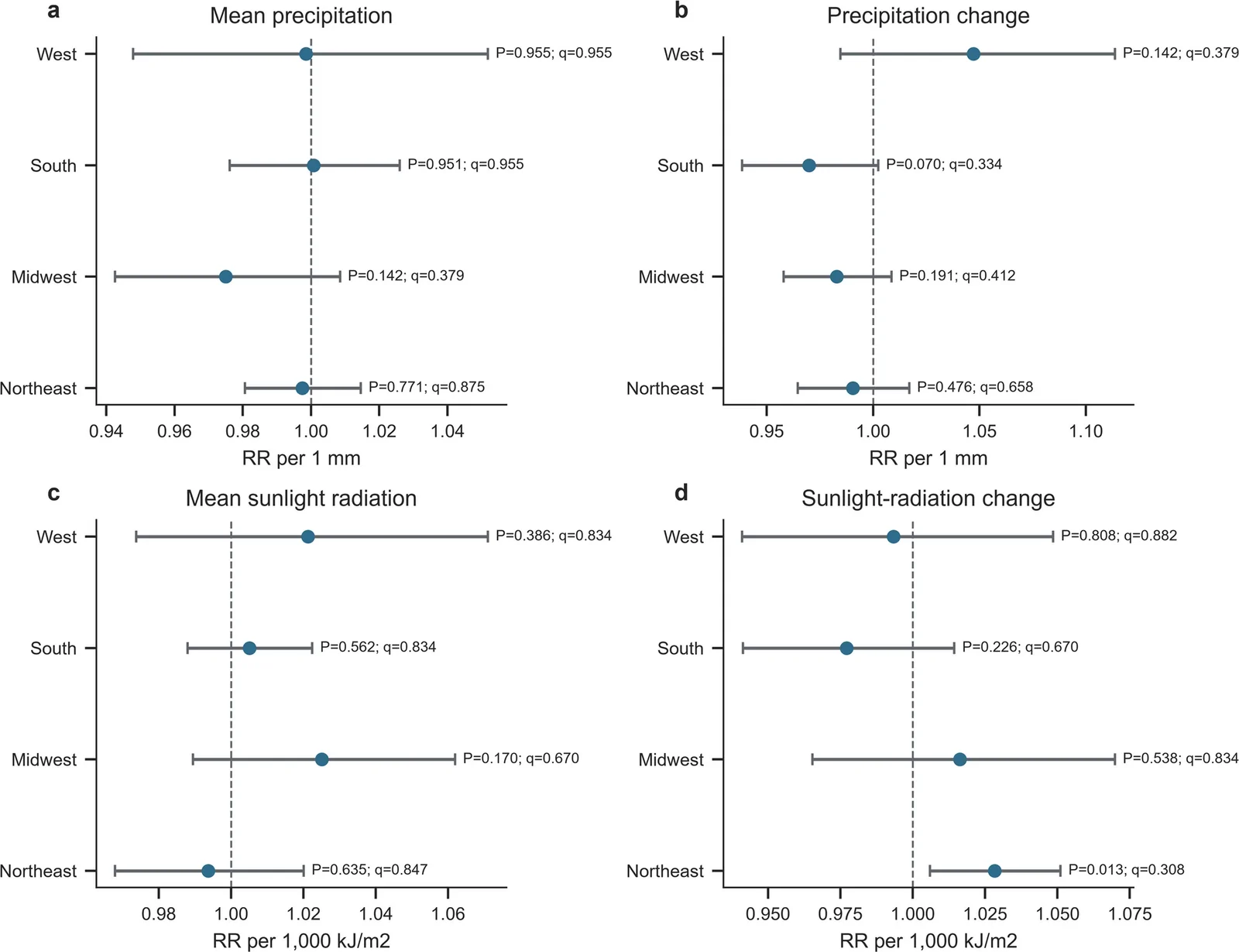
Adjusted precipitation and sunlight-radiation associations. Region-specific cumulative lag 0–3 Poisson models estimated RRs for mean precipitation (**a**), inter-month precipitation change (**b**), mean sunlight radiation (**c**), and inter-month sunlight-radiation change (**d**). Models included calendar-month fixed effects, a centered natural cubic spline of time, a log-population offset, and Newey-West covariance estimates. Points and bars are RRs and 95% CIs. Each panel reports nominal and BH-adjusted *P* values. BH, Benjamini-Hochberg; CI, confidence interval; RR, rate ratio

Cumulative lag 0–3 models showed significant positive associations for Northeast sunlight radiation change (RR, 1.028, 95% CI, 1.004–1.053) and Midwest mean sunlight radiation (RR, 1.025, 95% CI, 1.002–1.049). Restricted cubic spline analyses identified significant overall associations in three of four curves and nonlinear associations in the Northeast and Midwest (Fig. 12).

No sunlight lag-window estimate remained significant after BH correction; the minimum adjusted *P* value was 0.308. Detailed descriptive and single-lag sunlight results are shown in Figs. 10 and 11, respectively; cumulative adjusted models are shown in Fig. 12. The original sunlight time-series and monthly-profile panels are retained together in Figure S9, and additional lag-window and nonlinear results are shown in Figures S7 and S8.

### Integrated climate patterns from PCA

**Fig. 13:**
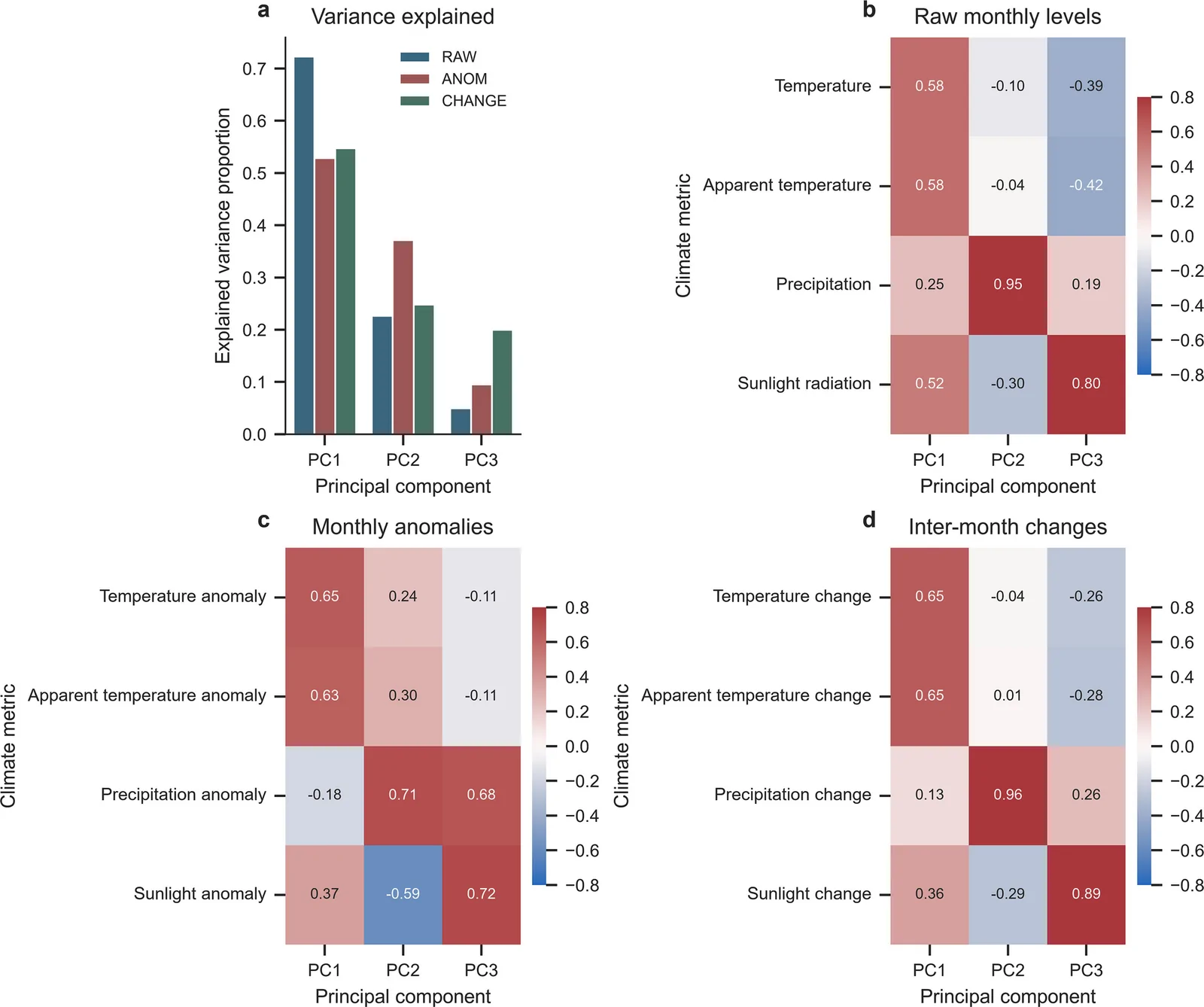
Principal component structure of regional climate metrics. Panel a shows variance explained by PC1-PC3 for raw monthly levels, monthly anomalies, and inter-month changes. Panels b-d show standardized loadings for temperature, apparent temperature, precipitation, and sunlight radiation. Positive and negative loadings indicate the direction of each metric along the component axis. PC, principal component

**Fig. 14:**
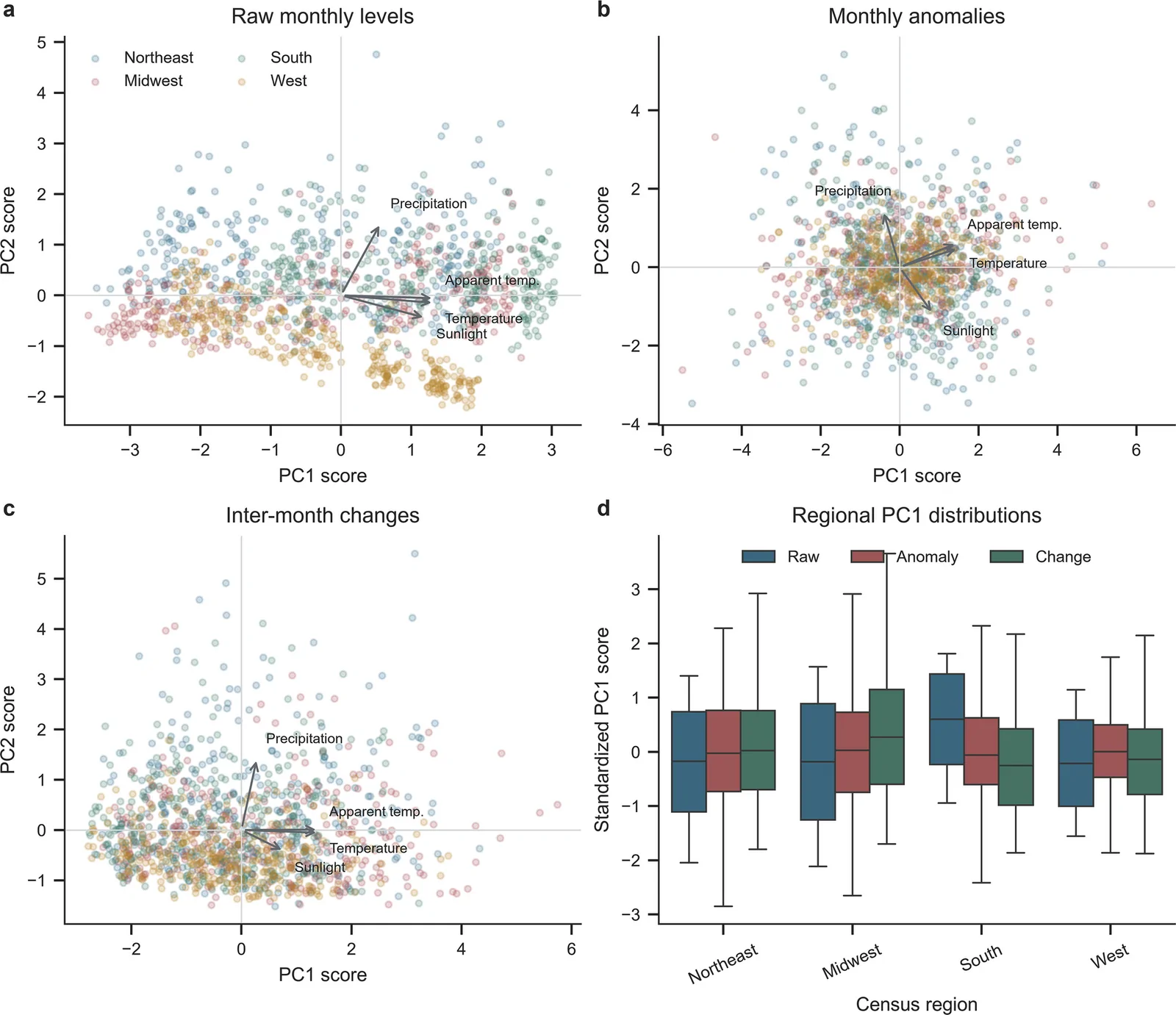
PCA biplots and regional PC1 score distributions. Biplots display monthly PC1-PC2 scores and variable-loading vectors for raw climate levels (**a**), monthly anomalies (**b**), and inter-month changes (**c**); colors identify Census regions. Panel d compares standardized PC1 score distributions across regions for all three climate blocks. PCA used temperature, apparent temperature, precipitation, and sunlight radiation. PC, principal component

PCA summarized correlated climate metrics into interpretable climate axes (Figs. 13 and 14). In the raw monthly climate block, PC1 explained 72.3% of variance, and PC1-PC2 together explained 95.0%. Raw PC1 had strong positive loadings for apparent temperature (0.577), mean temperature (0.576), and sunlight radiation (0.524), with a smaller positive loading for precipitation (0.247), indicating a warm, high-apparent-temperature, high-sunlight climate axis. (Supplementary Table S6).

For monthly anomalies, PC1 explained 52.9% of variance and PC1-PC2 together explained 90.0%. Anomaly PC1 was driven mainly by temperature anomaly and apparent-temperature anomaly, with a positive sunlight loading and a negative precipitation loading. For inter-month changes, PC1 explained 54.8% of variance and was driven primarily by month-to-month changes in temperature and apparent temperature.

**Fig. 15:**
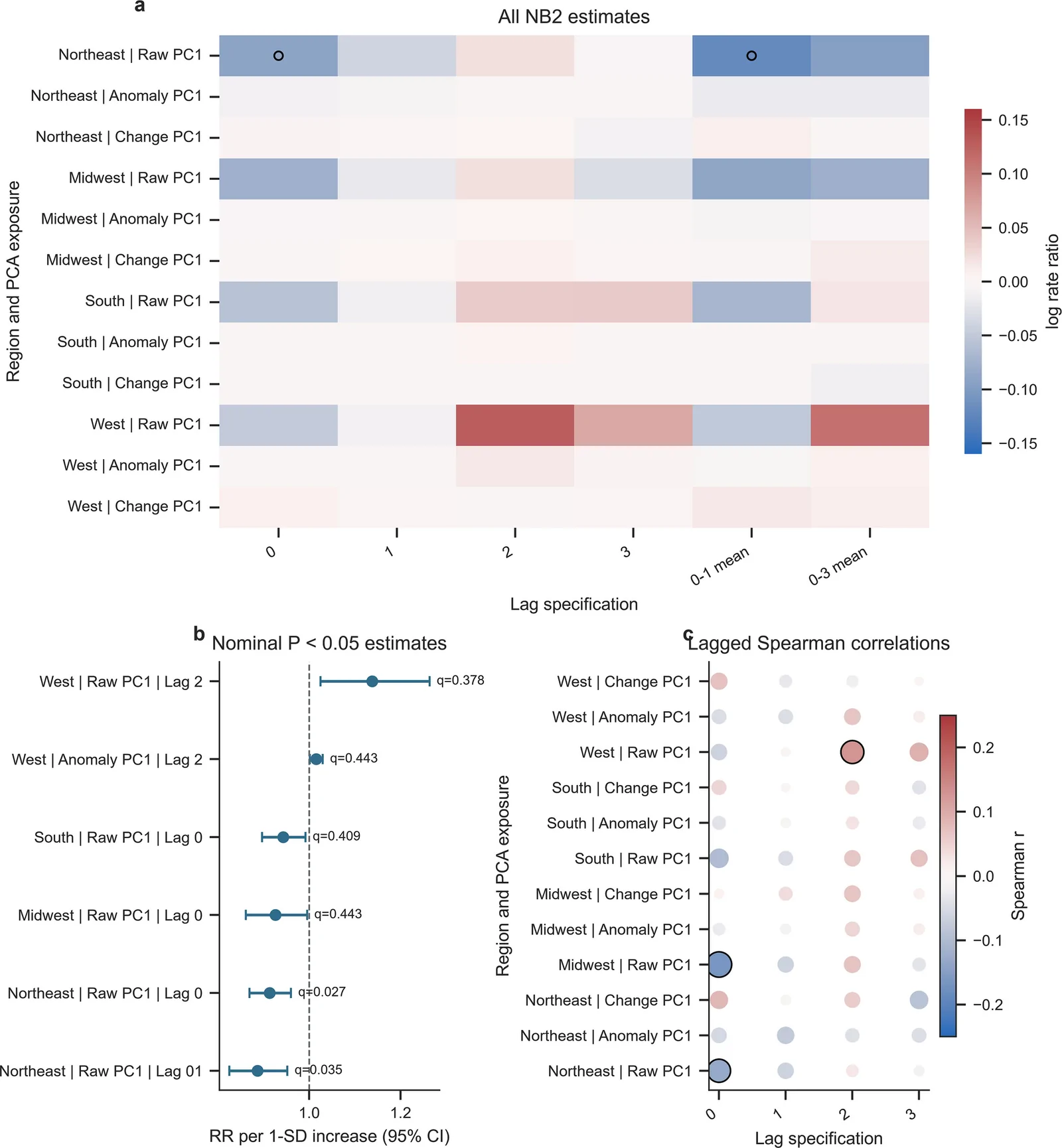
PCA climate dimensions and monthly Parkinson disease mortality. Panel a shows log RRs from 72 region-specific NB2 models for raw, anomaly, and change PC1 scores across six lag specifications; open circles identify BH-adjusted *P* < 0.05. Panel b shows the six estimates with nominal *P* < 0.05 and their adjusted *P* values. Panel c shows descriptive lagged Spearman correlations. NB2 models estimated alpha from the data, included a population offset and centered time spline, and used Newey-West covariance estimates. BH, Benjamini-Hochberg; CI, confidence interval; PC, principal component; RR, rate ratio

Negative binomial models for PC1 scores identified six nominally significant associations among 72 region-exposure-lag combinations. Raw PC1 was inversely associated with mortality in the Northeast at lag 0 (RR per 1-SD increase, 0.913; 95% CI, 0.869–0.960) and mean lag 0–1 (RR, 0.887; 95% CI, 0.825–0.952), in the Midwest at lag 0 (RR, 0.926; 95% CI, 0.861–0.995), and in the South at lag 0 (RR, 0.943; 95% CI, 0.897–0.992). In the West, raw PC1 showed a positive lag 2 association (RR, 1.138; 95% CI, 1.025–1.263), and anomaly PC1 showed a weaker positive lag 2 association (RR, 1.015; 95% CI, 1.001–1.030). PC1 restricted cubic spline models did not detect significant nonlinearity in any of the 12 curves (Fig. 15).

Poisson dispersion across the 72 PCA models had a median of 3.09 (range, 2.19–8.24), and NB2 had lower AIC than Poisson in all 72 comparisons. Of the six nominal associations, two Northeast raw PC1 estimates remained significant after BH correction: lag 0 (adjusted *P* = 0.027) and mean lag 0–1 (adjusted *P* = 0.035; Fig. 15). The remaining adjusted *P* values ranged from 0.378 to 0.443. Complete diagnostic and model-level results are provided in Supplementary Table S11.

## Discussion

In this national ecological time-series study, PD mortality among U.S. adults aged 65 years or older increased substantially from 1999 to 2024 and showed clear demographic, geographic, and meteorological patterning. The long-term rise was accompanied by higher mortality in men, steep age gradients, regional and state-level heterogeneity, and projected continued increases through 2035. The meteorological analyses added a seasonal and climate dimension to these mortality patterns. Temperature and apparent temperature showed the most consistent inverse associations with monthly crude PD mortality, especially in the Northeast and Midwest, whereas precipitation and sunlight radiation showed weaker and more heterogeneous associations. PCA further suggested that integrated climate dimensions, particularly warm and high-sunlight monthly climate patterns, were associated with lower mortality in selected regions and lag windows.

After forecast validation and multiplicity correction, the historical mortality increase was the most robust finding; long-horizon forecasts and most climate associations carried substantial uncertainty.

The observed increase in PD mortality is consistent with the broader epidemiologic expansion of PD as an age-related neurological disorder. Prior systematic reviews and meta-analyses have shown that PD is associated with higher mortality than the general population, and that excess mortality varies by age, disease duration, cognitive impairment, and comorbidity burden [31, 32]. Recent U.S. CDC WONDER analyses also reported increasing PD mortality among older adults before the most recent years included in the present study [33]. The present findings extend this evidence by integrating the 2021 to 2024 period, stratified geographic and demographic patterns, forward projections, and region-month meteorological analyses within one framework. The rise in AAMR suggests that the mortality burden is not explained by population aging alone. It may also reflect longer survival with advanced disease, improved recognition of PD on death certificates, shifts in care patterns, and increasing numbers of older adults living with complex neurological and systemic comorbidities.

Changes in diagnostic criteria and neurological awareness may increase recognition of PD, whereas symptomatic and advanced therapies may alter survival and patterns of late-stage care without eliminating fatal complications [51].

The demographic and geographic heterogeneity observed in this study is clinically plausible and important for surveillance. The higher mortality burden among men is consistent with epidemiologic evidence that PD incidence is higher in men than in women [38]. The steep age gradient likely reflects the combined influence of advanced neurodegeneration, frailty, aspiration risk, falls, immobility, autonomic dysfunction, and multimorbidity in very late life [34, 35, 49, 50]. Race, ethnicity, state, and regional differences should be interpreted with caution because mortality rates based on death certificates may reflect both disease burden and differential diagnosis, access to neurological care, coding practices, and end-of-life care. Prior work has documented disparities in advanced PD treatment use among insured older adults, which supports the possibility that access to specialty care and advanced therapies may contribute to geographic and sociodemographic differences in observed outcomes [37].

Differences in population composition, rurality, occupational history, diagnostic intensity, death-certification practice, and access to movement-disorder specialists, rehabilitation, long-term care, and palliative services may also contribute. The absence of significant global autocorrelation in 2024 argues against interpreting the latest map as a stable cluster pattern.

The forecasting results indicate that the public health burden of PD mortality is likely to continue increasing through 2035 if current trajectories persist. The higher projection under the pre-COVID scenario suggests that the pandemic period may have altered the observed mortality trajectory, although this finding should not be interpreted as a causal estimate of pandemic effects. Patients with PD may have been vulnerable during the COVID-19 period because of older age, frailty, respiratory dysfunction, reduced mobility, care disruption, and higher risks of severe outcomes after infection [35, 36]. The difference between the full-data and pre-COVID scenarios therefore highlights the need for sensitivity analyses when forecasting chronic disease mortality across periods with major health-system disruption.

Rolling-origin validation nevertheless showed underprediction and poor long-horizon interval coverage. The scenario contrast demonstrates sensitivity to the training period and does not isolate a causal COVID-19 effect; forecasts should therefore support scenario planning rather than precise resource targets.

The temperature findings are consistent with a broader environmental epidemiology literature showing that non-optimal temperature, temperature variability, and extreme heat or cold can increase mortality, particularly among older adults [7–9, 45–48]. In this study, warmer monthly temperature metrics were generally associated with lower PD mortality in descriptive and lagged models, especially in the Northeast and Midwest. This pattern may reflect winter-predominant mortality rather than a protective effect of heat itself. Prior PD-specific evidence has shown seasonal mortality patterns and heat wave-related morbidity and mortality signals, while a Japanese case-crossover analysis found associations between ambient temperature and nervous system disease-related mortality [15, 43, 44]. Taken together, these findings suggest that PD mortality may be sensitive to seasonal environmental conditions, but the direction and magnitude of risk probably depend on region, baseline climate, adaptive capacity, and the specific exposure window.

The inverse monthly association should not be interpreted as evidence that heat is protective; it may reflect winter mortality, infection, housing, behavior, adaptation, or residual seasonality.

Several mechanisms may explain why meteorological conditions could shape PD mortality at the population level. PD is frequently accompanied by autonomic dysfunction, impaired heart rate variability, and orthostatic hypotension, which may reduce physiological resilience to thermal stress [39, 40]. Respiratory dysfunction and aspiration pneumonia are also important in PD and are associated with higher hospital mortality, which may help explain winter-related vulnerability during periods of respiratory infection and reduced mobility [34, 35]. Inflammatory pathways may provide another link, since peripheral inflammatory cytokine abnormalities have been reported in PD and may interact with infection, cold stress, and systemic illness [41]. These pathways remain inferential in the present study because the data were ecological and did not contain individual-level clinical or biomarker information.

The precipitation, sunlight, and PCA findings should be interpreted as hypothesis-generating. Precipitation showed less consistent associations than temperature, which may reflect weaker direct biological relevance, regional differences in precipitation patterns, or residual confounding by season and other environmental conditions. Sunlight radiation was inversely correlated with crude mortality in descriptive analyses, but adjusted lagged models showed mixed associations. Prior meta-analytic evidence links vitamin D status, supplementation, outdoor work, and sunlight exposure with PD risk, although these studies address disease occurrence more than mortality [14, 42]. In the PCA analyses, raw monthly climate PC1 represented a warm, high-apparent-temperature, high-sunlight climate axis. Nominal inverse associations occurred in the Northeast, Midwest, and South, whereas nominal positive lag 2 associations occurred in the West. After BH correction, only Northeast raw PC1 at lag 0 and mean lag 0–1 remained significant. These findings suggest that integrated seasonal climate profiles may be more informative than single exposure metrics, but they do not establish which component of the climate axis is biologically responsible.

Most of these estimates attenuated after multiplicity correction, and PCA cannot identify which component of a correlated climate axis is causally responsible.

These findings have practical implications for public health planning and clinical care. PD mortality surveillance should consider not only long-term demographic aging but also seasonal and regional vulnerability. Older adults with PD, especially those with advanced disease, frailty, respiratory dysfunction, autonomic symptoms, or limited access to specialty care, may benefit from anticipatory seasonal planning. This could include winter respiratory infection prevention, fall and aspiration risk reduction, medication review, caregiver support, temperature-safe housing, and targeted outreach during periods of unusual cold, heat, or rapid temperature change. The projected rise through 2035 also suggests that health systems in high-burden regions may need to prepare for increasing demand in neurology, rehabilitation, long-term care, palliative care, and caregiver services.

Because long-horizon calibration was limited, these implications should be revisited as new mortality observations become available and should not be interpreted as precise demand forecasts.

### Strengths and limitations

This study has several strengths. It used a national mortality database covering 26 years, examined multiple demographic and geographic strata, projected future mortality, and linked monthly regional mortality with meteorological data from NASA Earthdata/NLDAS. The analysis also combined Joinpoint trend estimation, ETS forecasting, lagged Poisson models, restricted cubic splines, and PCA-based climate summaries.

Several limitations should be acknowledged. First, death certificate data may misclassify PD as an underlying cause of death, and certification practices may vary across states and over time.

Second, the ecological design precludes individual-level causal inference and does not permit adjustment for disease duration, medications, socioeconomic status, institutionalization, comorbidities, indoor temperature, infection, or individual mobility. Third, monthly Census-region exposures may obscure short-term extremes, within-region spatial heterogeneity, and personal exposure conditions. Area-weighted regional averages do not represent where older adults live, indoor temperature, housing quality, personal mobility, or individual exposure. Such ecological exposure misclassification may attenuate, distort, or create associations, and regional estimates cannot be transferred to individual risk [58]. Residual confounding by infection, air pollution, socioeconomic conditions, healthcare access, institutionalization, and comorbidity remains possible.

Fourth, multiple regions, exposures, and lag windows were evaluated. BH correction reduced the number of supported associations, and nonlinear analyses remained exploratory. The 0–3-month lag was biologically plausible but not uniquely determined, although 0–1 and 0–6 sensitivity analyses were reported. Finally, forecasts were sensitive to the training period and showed low long-horizon coverage; they should be updated as new data accrue.

## Conclusion

PD mortality among older adults in the United States increased markedly from 1999 to 2024. Demographic and geographic differences were pronounced, whereas long-horizon forecasts and ecological climate associations carried considerable uncertainty. Continued surveillance and regularly updated, locally calibrated planning are warranted.

## Supplementary Information

Supplementary Material 1.

Supplementary Material 2.

Supplementary Material 3.

Supplementary Material 4.

Supplementary Material 5.

Supplementary Material 6.

Supplementary Material 7.

Supplementary Material 8.

Supplementary Material 9.

Supplementary Material 10.

Supplementary Material 11.

## Data Availability

Mortality data are publicly available from CDC WONDER. Meteorological data are publicly available from NASA Earthdata/NLDAS. The numbered Supplementary Tables S1-S11 and Supplementary Data S1-S2 are provided in Supplementary_Revision_Analyses.xlsx, with accompanying machine-readable CSV files for audit and reuse.
