# Supplementary material for: Spatiotemporal dynamics and climate correlates of Parkinson disease mortality among older U.S. adults: national trends and forecasts to 2035
Source: BMC Neurol. 2026 Aug 12;26:712. doi: 10.1186/s12883-026-05248-y (PMC13746532; doi:10.1186/s12883-026-05248-y)
Supplement: Supplementary file 1 [file 12883_2026_5248_MOESM10_ESM.docx]

**STROBE Checklist**

Checklist completed for the manuscript titled "Parkinson Disease Mortality Among Older Adults in the United States, 1999-2035: Spatiotemporal Trends, Forecasts, and Meteorological Correlates". Section names are provided instead of page numbers and can be replaced with page and line numbers after final journal formatting.

| **Item** | **STROBE domain** | **Recommendation** | **Location in manuscript** | **Response** |
| --- | --- | --- | --- | --- |
| 1 | Title and abstract | Identify the observational design and provide a balanced summary of what was done and found. | Title and Abstract | The abstract states that this was an ecological time-series study and summarizes data sources, methods, results, and conclusions. |
| 2 | Background/rationale | Explain the scientific background and rationale for the investigation. | 1 Introduction | The introduction describes rising PD burden, mortality surveillance needs, and the environmental rationale. |
| 3 | Objectives | State specific objectives and any prespecified hypotheses. | 1 Introduction | The final introduction paragraph states the study objectives and analytic approach. |
| 4 | Study design | Present key elements of the study design early in the paper. | 2.1 Study design and data sources | The design is described as an ecological time-series study. |
| 5 | Setting | Describe setting, locations, dates, and periods of data collection. | 2.1 Study design and data sources | The study covers U.S. adults aged 65 years or older from January 1999 through December 2024. |
| 6 | Participants | Describe eligibility criteria and sources of study data. | 2.1 Study design and data sources | The study used aggregate CDC WONDER mortality records for adults aged 65 years or older with PD as the underlying cause of death. Individual participant selection was not applicable. |
| 7 | Variables | Define outcomes, exposures, predictors, confounders, and effect modifiers. | 2.2 Mortality outcomes and rate definitions; 2.5 Single meteorological exposure analyses; 2.6 Principal component analysis of climate metrics | Mortality outcomes, demographic strata, geographic strata, meteorological exposures, lags, and PCA metrics are defined. |
| 8 | Data sources and measurement | For each variable, provide data sources and methods of assessment. | 2.1 Study design and data sources; 2.2 Mortality outcomes and rate definitions | Mortality data came from CDC WONDER and meteorological data came from NASA Earthdata/NLDAS. |
| 9 | Bias | Describe efforts to address potential sources of bias. | 2 Methods; 4 Discussion | The analysis used standardized public mortality data, stratification, lagged models, spline adjustment for time trend, and discusses ecological and death-certificate limitations. |
| 10 | Study size | Explain how the study size was arrived at. | 2.1 Study design and data sources | All eligible national aggregate mortality records for 1999 to 2024 were included. A separate sample-size calculation was not applicable. |
| 11 | Quantitative variables | Explain how quantitative variables were handled in the analyses. | 2.2 Mortality outcomes and rate definitions; 2.5 Single meteorological exposure analyses; 2.6 Principal component analysis of climate metrics | Rates, exposure lags, spline terms, offsets, and standardized PCA scores are described. |
| 12 | Statistical methods | Describe statistical methods, subgroup analyses, missing data handling, and sensitivity analyses. | 2.3 Joinpoint trend analysis; 2.4 Forecasting analysis; 2.5 Single meteorological exposure analyses; 2.6 Principal component analysis of climate metrics; 2.7 Statistical software and ethics | Joinpoint regression, ETS forecasting, Poisson models, restricted cubic splines, PCA, negative binomial models, software, and significance thresholds are reported. |
| 13 | Participants or observations | Report numbers of observations at each stage of the study. | 3 Results; Table 1 | Annual deaths and subgroup mortality outputs are reported. Individual participant flow was not applicable for aggregate mortality data. |
| 14 | Descriptive data | Give characteristics of study participants or observations. | 3 Results; Table 1; Figure 2 | Deaths, rates, age groups, sex, race and ethnicity, region, and state patterns are summarized. |
| 15 | Outcome data | Report numbers of outcome events or summary outcome measures. | 3 Results; Table 1 | PD deaths, AAMRs, crude rates, and forecasted rates are reported. |
| 16 | Main results | Give unadjusted and adjusted estimates with precision where appropriate. | 3 Results; Figures 2-7 | Trend estimates, forecasts, correlations, rate ratios, confidence intervals, and spline results are reported. |
| 17 | Other analyses | Report other analyses such as subgroup, sensitivity, and exploratory analyses. | 3 Results; Figures 3-7 | The study reports subgroup forecasts, regional lagged analyses, restricted cubic spline analyses, and PCA-based climate analyses. |
| 18 | Key results | Summarize key results with reference to study objectives. | 4 Discussion | The opening Discussion paragraph summarizes the central findings. |
| 19 | Limitations | Discuss study limitations and sources of potential bias. | 4 Discussion | Limitations include death-certificate misclassification, ecological design, exposure aggregation, multiple testing, and forecast uncertainty. |
| 20 | Interpretation | Provide cautious overall interpretation considering objectives, limitations, multiplicity, and related evidence. | 4 Discussion | The Discussion interprets findings in relation to prior PD, mortality, and environmental epidemiology literature. |
| 21 | Generalisability | Discuss the external validity of the study results. | 4 Discussion | The Discussion notes national scope while limiting inference to aggregate U.S. mortality and regional meteorological exposure patterns. |
| 22 | Funding | Give the source of funding and the role of funders. | 6 Declarations | The manuscript states that no specific funding was reported and notes that this should be updated if funding applies. |
